# Accuracy of Genomic Prediction in Switchgrass (*Panicum virgatum* L.) Improved by Accounting for Linkage Disequilibrium

**DOI:** 10.1534/g3.115.024950

**Published:** 2016-02-10

**Authors:** Guillaume P. Ramstein, Joseph Evans, Shawn M. Kaeppler, Robert B. Mitchell, Kenneth P. Vogel, C. Robin Buell, Michael D. Casler

**Affiliations:** * Department of Agronomy, University of Wisconsin-Madison, WI 53706; † Department of Energy Great Lakes Bioenergy Research Center, Michigan State University, East Lansing, MI 48824; ‡ Department of Plant Biology, Michigan State University, East Lansing, MI 48824; § Department of Energy Great Lakes Bioenergy Research Center, University of Wisconsin-Madison, WI 53706; ** Grain, Forage, and Bioenergy Research Unit, Agricultural Research Service, United States Department of Agriculture, University of Nebraska, Lincoln, NE 68583-0937; †† Agricultural Research Service, United States Department of Agriculture, Madison, WI 53706

**Keywords:** genomic selection, linkage disequilibrium, exome capture, bioenergy, *Panicum virgatum* L, GenPred, Shared data resource

## Abstract

Switchgrass is a relatively high-yielding and environmentally sustainable biomass crop, but further genetic gains in biomass yield must be achieved to make it an economically viable bioenergy feedstock. Genomic selection (GS) is an attractive technology to generate rapid genetic gains in switchgrass, and meet the goals of a substantial displacement of petroleum use with biofuels in the near future. In this study, we empirically assessed prediction procedures for genomic selection in two different populations, consisting of 137 and 110 half-sib families of switchgrass, tested in two locations in the United States for three agronomic traits: dry matter yield, plant height, and heading date. Marker data were produced for the families’ parents by exome capture sequencing, generating up to 141,030 polymorphic markers with available genomic-location and annotation information. We evaluated prediction procedures that varied not only by learning schemes and prediction models, but also by the way the data were preprocessed to account for redundancy in marker information. More complex genomic prediction procedures were generally not significantly more accurate than the simplest procedure, likely due to limited population sizes. Nevertheless, a highly significant gain in prediction accuracy was achieved by transforming the marker data through a marker correlation matrix. Our results suggest that marker-data transformations and, more generally, the account of linkage disequilibrium among markers, offer valuable opportunities for improving prediction procedures in GS. Some of the achieved prediction accuracies should motivate implementation of GS in switchgrass breeding programs.

Genomic selection (GS) is the use of genome-wide marker information to predict genotype performance in breeding programs. Predictions in GS should be sufficiently accurate so that it is economically more viable to select individuals based solely on markers, rather than phenotypic measurements (Muir 2007; Lorenz *et al.* 2011; Riedelsheimer and Melchinger 2013). In switchgrass (*Panicum virgatum* L.), a perennial forage crop, this potential advantage derives from the fact that phenotypic measurements generally require 2–3 yr of field testing (one establishment year and 1–2 yr of trial; Casler and Brummer 2008), whereas acquiring genome-wide marker information would require less than a year (Resende *et al.* 2014). The US Department of Agriculture and the US Department of Energy intend to make switchgrass a principal source of biofuel in the US by 2030, so as to meet the goal of displacing 30% of petroleum use with biofuel (Sanderson *et al.* 1996; Perlack *et al.* 2005). However, strong and rapid genetic gains for biomass yield to approximately 20 Mg/ha are required to meet this goal (Perlack *et al.* 2005). Therefore, in the case of switchgrass breeding, GS is a technology that is not only economically attractive, but also strategically useful.

The first genomic prediction procedure, introduced by Meuwissen *et al.* (2001)—ridge regression-BLUP (RR-BLUP), equivalent to genomic BLUP (GBLUP) (Hayes *et al.* 2009)—assumed an infinitesimal genetic model (*i.e.*, all loci in linkage equilibrium with additive effects of equal variance), and a homogeneous genetic architecture throughout individuals. Since then, improvements in genomic prediction models have been made, most notably through the account of (i) marker-effect heteroscedasticity, *i.e.*, differential variances of marker effects (Meuwissen *et al.* 2001; Park and Casella 2008; Erbe *et al.* 2012; Shen *et al.* 2013); (ii) nonlinear marker effects and interactions between markers (Gianola and van Kaam 2008; Akdemir and Jannink 2015); (iii) genotype-by-environment interactions (Burgueño *et al.* 2012; Guo *et al.* 2013; Heslot *et al.* 2014); (iv) correlation among traits of interest (Calus and Veerkamp 2011; Jia and Jannink 2012); and (v) population heterogeneity (Harris and Johnson 2010; Makgahlela *et al.* 2013; Karoui *et al.* 2012; Isidro *et al.* 2015).

Marker-data transformations have previously been used in GS to account for marker-effect heteroscedasticity, with weights on marker variables reflecting the significance of their relationship with the outcome of interest (de los Campos *et al.* 2013; Su *et al.* 2014; He *et al.* 2015). Transformations on marker data have also been used to account for redundancy in marker information due to linkage disequilibrium (LD), through principal component analysis (PCA) (Long *et al.* 2011); or weights on marker variables; so as to reflect the degree of tagging of loci by markers (Speed *et al.* 2012; Nishio and Satoh 2015). However, to date, few empirical studies in GS have been conducted to assess the usefulness of preprocessing marker data in order to account for LD.

In this study, we assessed the possibility of producing reliable predictions for GS in switchgrass. Our data consisted of relatively few observations on two distinct populations. A total of 247 individuals were genotyped by exome capture sequencing, and evaluated for important agronomic traits: biomass yield, plant height, and heading date. We assessed various types of prediction procedures, which differed from the standard procedure (GBLUP on nontransformed marker data) not only by the prediction model—which might account for heteroscedasticity and/or nonlinearity of marker effects—but also by the type of marker-data transformation—which might account for LD among markers. We also examined the potential of learning schemes (training-set designs) for improving genomic prediction procedures. While multiple-trait models may be useful to account for genotype-by-environment interactions, correlation among traits and/or population heterogeneity, such models were not found useful here because they either were not statistically efficient enough, or failed to effectively fit the data, certainly as a result of our small sample sizes.

## Materials and Methods

### Populations assayed

Genomic selection (GS) in switchgrass was studied in two tetraploid populations. The first population comprised 137 half-sib (HS) families developed from WS4U, an upland-ecotype germplasm pool of 162 plants (Casler *et al.* 2006). The HS families were the progeny of genotypes produced in cycle 2 (C2) of selection on WS4U for high biomass yield (Casler 2010; Casler and Vogel 2014). The second population comprised 110 HS families developed from the cultivar Liberty, which is a stabilized lowland-upland hybrid cultivar (Vogel *et al.* 2014; Casler and Vogel 2014). The HS families were developed from the Liberty population by an additional breeding generation. They were the progeny of the genotypes selected in C2 for high biomass yield, excellent winter survival, excellent spring greenup, and no apparent diseases using the among and within family breeding method. In both populations, the selected C2 genotypes were polycrossed in isolation. The two populations, hereafter referred to as WS4U-C2 and Liberty-C2, were tested in two locations in the United States: Arlington (WI) and Mead (NE) in 2012, 2013, and 2014. Families were assayed in a row-plot trial and replicated in a randomized complete block design (RCBD), with four family replicates for WS4U-C2, and three family replicates for Liberty-C2. There were up to five HS in each family replicate, with different HS between replicates (there was no vegetative propagation of individual plants). Rows were spaced 0.9 m apart, and plants were spaced 0.45 m apart within rows. Plots were established from greenhouse-grown seedlings in May 2011, and fertilized with 110 kg N ha^–1^ in May of 2012 and 2013.

### Phenotypic data and mixed-model analyses

In this study, trait measurements at different locations were considered different outcomes. There were six outcomes: dry matter yield (DMY), plant height (PH), and heading date (HD) in WI or NE. HD was scored on each individual plant as day-of-year when half of the panicles of a plant had fully emerged from the boot. PH was measured on each individual plant from the ground to the top of the tallest tiller after growth had ceased in late September. DMY was determined by harvesting each row plot with a flail harvester at a 10-cm cutting height, adjusted for moisture concentration using a 400-g sample of harvested tissue dried for 7 d at 60°. Outcomes were measured in 2012 and 2013, with the exception of HD in WI, which was scored in 2013 and 2014 for WS4U-C2 and in 2012, 2013, and 2014 for Liberty-C2 (Table 1).

**Table 1:** Description of trait measurements for WS4U-C2 and Liberty-C2 in WI and NE

Population	Location	Trait	Years of Trial	Range	Mean	SD	Reliability (SD)
WS4U-C2	WI	PH	2012 2013	62–286	160	41	0.69 (0.014)
		HD	2013 2014	180–219	196	6	0.76 (0.011)
		DMY	2012 2013	73–1158	399	180	0.10 (0.029)
	NE	PH	2012 2013	60–252	170	24	0.75 (0.019)
		HD	2012 2013	171–232	199	11	0.74 (0.013)
		DMY	2012 2013	84–1224	490	190	0.45 (0.065)
Liberty-C2	WI	PH	2012 2013	62–272	183	30	0.61 (0.045)
		HD	2012 2013 2014	189–242	216	8	0.76 (0.038)
		DMY	2012 2013	18–1169	455	216	0.21 (0.059)
	NE	PH	2012 2013	65–298	216	24	0.67 (0.0038)
		HD	2012 2013	200–275	232	11	0.66 (0.0036)
		DMY	2012 2013	377–1504	861	207	0.53 (0.04)

Population: WS4U-C2 (collection of upland ecotypes) or Liberty-C2 (cross between upland and lowland ecotypes). Location: Arlington (WI) or Mead (NE). Units for Range, Mean and SD are centimeter, day of the year, and gram per plant, for PH, HD, and DMY, respectively. Reliability: inferred squared correlation between a true family effect and its BLUP from the mixed models presented in *Material and Methods*. PH, plant height; HD, heading date; DMY, dry matter yield.

For PH and HD outcomes, measured on an individual-plant basis, the following linear mixed model was fitted:

$$y_{ijkl}=\mu +g_{i}+b_{j}+t_{k}+{\left(g\times b\right)}_{ij}+{\left(g\times t\right)}_{ik}+{\left(b\times t\right)}_{jk}+{\left(g\times b\times t\right)}_{ijk}+\varepsilon_{ijkl}$$

where μ is the grand mean; $g_{i}$, $b_{j}$, and $t_{k}$ are the random effects of HS family i, block j, and year k, respectively; × indicates interactions; $\varepsilon_{ijkl}$ are residuals. For each term, the corresponding effects were modeled as independent and identically normally distributed. For PH (in both locations), an additional term $\mathrm{plo}t_{ij}\;\sim \text{ Normal}\left(0,\left(\Sigma_{r}⊗\Sigma_{c}\right)\sigma_{plot}^{2}\right)$ was included in the model (on the basis of a lower Bayesian information criterion), where $\Sigma_{r}⊗\Sigma_{c}$ is the Kronecker product of the first-order autoregressive covariance matrices on rows and on columns, respectively.

For DMY outcomes, measured on a plot basis, the following linear mixed model was fitted:

$$y_{ijk}=\mu +g_{i}+b_{j}+t_{k}+{\left(g\times b\right)}_{ij}+{\left(g\times t\right)}_{ik}+{\left(b\times t\right)}_{jk}+e_{ijk}$$

where effects are as described above, except for $e_{ijk}$, which is the pooled error of plot ij in year k. The linear mixed models described here were all fitted using ASREML-R (Butler *et al.* 2007).

The predicted HS-family effects are best linear unbiased predictions (BLUPs) of the transmitting abilities of maternal parents. On the one hand, BLUPs have the property of being shrunk toward their mean (zero) differentially, depending on the relative amount of information available for their computation. As a result, estimates of marker effects based on BLUPs tend to be distorted compared to the estimates based directly on phenotypes (Garrick *et al.* 2009), which can be problematic in inferential studies such as QTL analyses, especially if reliabilities of BLUPs are highly variable among genotypes. So, in quantitative genetic analyses, it has been recommended to deregress BLUPs for subsequent use in weighted regression models, accounting for differential levels of uncertainty in the deregressed-BLUP estimates, rather than using nonweighted regression models on BLUPs directly. On the other hand, BLUPs are generally more accurate estimates of the true values, and the approach based on BLUPs does not rely on (possibly suboptimal) weights in regression. Importantly, Guo *et al.* (2010) showed, in simulation studies, that GS models based on BLUPs predicted true performance of genotypes as, or more, accurately than weighted GS models based on “daughter yield deviations”, equivalent to deregressed BLUPs (even when strong differences in available information were simulated), which suggests that BLUPs are acceptable alternatives to their deregressed counterpart as response variables in predictive studies. Consequently, here we chose not to deregress HS-family BLUPs, and use them directly as response variables for training and validating GS models. Nonetheless, all methods used in this study can be adapted to accommodate deregressed BLUPs with differential weights on observations, if needed.

The raw phenotypic data and the matrix of HS-family BLUPs are available online as Supplemental Material, File S1 and File S2, respectively, and from http://dfrc.wisc.edu/sniper/.

### Marker data and quality control

Exome capture sequencing of HS-family maternal parents was performed using the Roche-Nimblegen protocol for preparation of SeqCap EZ Developer libraries using the Roche-Nimblegen probeset ‘120911_Switchrass_GLBRC_R_EZ_HX1’ as described previously (Evans *et al.* 2014, 2015). Capture was performed on the 247 individuals from WS4U-C2 and Liberty-C2, and sequencing was performed on the Illumina HiSequation 2000 platform, generating 150-nt paired-end reads. Initial quality control was performed using FastQC (v0.10.0; http://www.bioinformatics.babraham.ac.uk/projects/fastqc/). PCR primers, adapter sequences, and bases with quality scores below 20 were trimmed using Cutadapt (v1.1; https://code.google.com/p/cutadapt/). Reads with lengths shorter than 35 nt were discarded. Cleaned reads were aligned to the hardmasked *P. virgatum* v1.1 reference genome (http://phytozome.jgi.doe.gov/pz/portal.html#!info?alias=Org_Pvirgatum) using BowTie v0.12.7 (Langmead *et al*. 2009). Unanchored contigs were assigned to scaffolds (ChrUn1-ChrUn15) for more efficient alignment (Evans *et al.* 2015). Unique alignments were required, and only a single mismatched nucleotide was permitted in the first 35 bases of the read. Read alignments meeting the alignment criteria were processed using the index, sort, merge (default parameters), and mpileup (-BD –C 0 options) functions of the SAMTools package v0.1.18 (Li *et al.* 2009). Counts of reads corresponding to reference and alternate alleles were generated for each individual in the WS4U-C2 and Liberty-C2 populations at a subset of sites (2,179,164 loci; HapMap v2) previously determined to be polymorphic using exome capture sequencing data from two diversity panels, the Northern Switchgrass Panel (Evans *et al.* 2015) and a southern switchgrass panel (C. Brummer, unpublished data). Then, marker genotypes were called, assuming disomic inheritance of tetraploid switchgrass based on previous genetic-mapping studies (Okada *et al.* 2010; Li *et al.* 2014). To infer marker genotypes while effectively accounting for genotype-calling uncertainty, read counts were converted to expected allelic dosages (values between 0 and 2 for the number of copies of the alternate allele); using the algorithm of Martin *et al.* (2010), fitted on each population separately. The algorithm of Martin *et al.* (2010) estimates the sequencing/alignment error rate and the population allele frequency for each marker separately, using an Expectation-Maximization (EM) algorithm. Then, for each combination of marker and individual, the posterior probability of each allelic dosage given the read-count data is obtained by Bayes’ rule, assuming Hardy-Weinberg equilibrium (HWE) to derive the prior probability of each allelic dosage, and a binomial distribution of read type (reference/alternate) to derive the likelihood of each allelic dosage. Expected allelic dosages were computed as the sum of possible allelic dosages weighted by their posterior probability for each combination of individual and marker.

In the resulting matrix of expected allelic dosages, marker variables were then filtered for (i) proportion of missing values (strictly lower than 5%); (ii) polymorphism (minor allele frequency across populations strictly greater than 1/2N, and variance higher than 2(1/2N)(1−1/2N), with N the total number of genotypes across populations); (iii) HWE within each population (p-value for HWE, based on a χ^2^-test, strictly higher than 10^−4^ for each population considered individually); and (iv) availability of genomic-location information (available information on chromosome and position from the reference genome sequence, and annotation of *P. virgatum* v1.1; DOE-JGI, http://phytozome.jgi.doe.gov/). The resulting matrix M contained expected allelic dosages at $q^{*}=141,030$ selected markers across populations, $q^{*}=108,077$ in WS4U-C2 only and $q^{*}=79,543$ in Liberty-C2 only.

To characterize LD between markers, we used the correlation matrix R consisting of Pearson correlation coefficients between allelic dosages: $R_{jj^{’}}=Cor(M_{j},M_{j^{’}})$, where indexing on M refers to columns. For matters of efficiency, the $q^{*}\times q^{*}$ R matrix was made block-diagonal, with blocks corresponding to chromosomes (i.e., only local LD was accounted for, through R). This assumed sparsity in marker correlations allowed us to compute R with reasonable costs in time and memory, while potentially reducing noise in the estimations by assuming zero correlation between markers from different chromosomes. Positive-definiteness of R (which implies invertibility) was ensured using the modified projection algorithm of Higham (2002) from the nearPD function of the R package Matrix.

Following Speed *et al.* (2012), for any marker $M_{j}$ we define the degree of (local) tagging as the sum of squared correlations involving $M_{j}$ and any other marker within the same chromosome, *i.e.*, $\underset{j^{’}}{\sum }R_{jj^{’}}^{2}$, with $j^{’}$ indexing markers on the same chromosome as $M_{j}$’s. This metric is supposed to depict the redundancy in information at $M_{j}$, as reflected by R.

The matrix M for markers selected across both populations is available online as a supplementary file (File S3, with values rounded to the fifth decimal digit) and from http://dfrc.wisc.edu/sniper/ (with nonrounded values).

### Prediction procedures

For each possible combination of population and outcome, we evaluated prediction procedures with respect to four components: (i) population learning scheme—set of parent genotypes to include for training; (ii) environment learning scheme—set of locations to include for training; (iii) marker-data transformation—type of transformation on the marker data used to produce marker features; and (iv) prediction model—method used to generate predictions on the outcome based on marker features. In this study, emphasis was placed on the last two components.

#### Prediction models:

The standard statistical model for prediction was genomic BLUP (GBLUP; Habier *et al.* 2007; Hayes *et al.* 2009). For a sample of n instances and q marker features, we define GBLUP as follows:

g=μ+Zu+e

where $g=\left\{g_{i}\right\}$ is the n-vector of HS-family BLUPs; μ is the n-vector of grand mean; Z is the n × m design matrix attributing the n observations to m parent genotypes; $u\;\sim \text{ Normal}(0,K\sigma_{u}^{2}$), K being the m × m genomic relationship matrix derived from marker features as $K\propto XX^{T}$, with X the m × q matrix of marker features; $e\;\sim \text{ Normal}(0,\;I\sigma_{e}^{2}$), with I the identity matrix. As explained in the next subsection, the marker features in X were not the expected allelic dosages, *i.e.*, X≠M. The normalizing factor in K was the sum of sample variances over marker features.

The GBLUP model is equivalent to the RR-BLUP model, where the assumptions of an infinitesimal genetic model are made: effects of marker features are assumed to be additive, linear, homoscedastic (*i.e.*, having equal variance), and independent (which implies no LD between markers). To accommodate genetic architectures that strongly deviate from the infinitesimal model, we considered eight additional models that were heteroscedastic and/or nonlinear.

Heteroscedastic models were GBLUP-wG, GBLUP-sG, BayesA, and BayesB. The GBLUP-wG model, first used by de los Campos *et al.* (2013), consisted in weighting marker features by $-log_{10}(p)$, where *p* is the p-value for the effect of a marker feature on the outcome of interest. GBLUP-sG is a variation from GBLUP-wG, where are included in the model only the marker features with a false discovery rate (FDR) for their correlation with the outcome lower than some threshold, determined by tuning; the FDR was calculated using the qvalue package in R (Storey and Tibshirani 2003). BayesA and BayesB are Bayesian linear regression models, introduced by Meuwissen *et al.* (2001), which have the following specification:

g=μ+Xb+e

where g, μ, X are as described above; $b\;\sim \text{ Normal}(0,\;I\sigma_{b}^{2}$). In BayesA, $\sigma_{b}^{2}\;\sim \;\chi^{-2}(df_{b},S_{b}^{2})$. In BayesB, $\sigma_{b}^{2}=0$ with probability π, and $\sigma_{b}^{2}\;\sim \;\chi^{-2}(df_{b},S_{b}^{2})$ with probability 1−π; π was chosen to follow a Beta(0.2, 1.8) in order to reflect relatively sparse distributions of causal variants across the genome while allowing uncertainty about π. In both BayesA and BayesB, $\;S_{b}^{2}\;\sim \;\text{Gamma}(r_{b},s_{b})$, and $e\;\sim \text{ Normal}(0,I\sigma_{e}^{2}$), with $\sigma_{e}^{2}\;\sim \;\chi^{-2}(df_{e},S_{e}^{2})$. The hyperparameters $df_{b}$, $r_{b}$, s_b, $df_{e}$, and $S_{e}^{2}$ were set through the heuristics described in Pérez and de los Campos (2014), based on a prior estimation of the proportion of variance explained by the model, which was here chosen to be $\frac{\sigma_{u}^{2}}{\sigma_{u}^{2}+\sigma_{e}^{2}}$ from a GBLUP model with an update on marker effects from the heteroscedastic effects model (HEM) of Shen *et al.* (2013). BayesA and BayesB were fitted by a Gibbs sampling algorithm with 5000 burn-in iterations, then 15,000 iterations for actual sampling of parameter values.

The one nonlinear model that we assayed was the reproducing kernel Hilbert space (RKHS) model described by Gianola and van Kaam (2008). In the implementation recommended by these authors, the RKHS model is made equivalent to the GBLUP model, where pairwise relationship coefficients in K are replaced by an appropriate nonlinear function of pairwise distances. The pairwise distances were Euclidean distances based on marker features, scaled by the maximum distance over pairs of individuals; the nonlinear function was the Gaussian kernel, with its scale parameter determined by tuning.

To account for both heteroscedasticity and nonlinearity, we extended the RKHS model to RKHS-wG and RKHS-sG, where marker features were weighted, as described above for GBLUP-wG and GBLUP-sG. One last heteroscedastic and nonlinear model that we considered was Random Forest (RF), which is a machine-learning method that combines results from several regression (or classification) trees, fitted to different variations of the data—bootstrap samples of instances and random subsets of features (Breiman 2001). The RF model was fitted with 200 trees, bootstrap samples of size n, and subsets of q/3 features.

Tuning for the scale parameter in RKHS (and its extensions -wG and -sG), and the FDR threshold in GBLUP-sG or RKHS-sG, was performed through minimization of the generalized-cross-validation criterion (GCV; Golub *et al.* 1979; Gu and Ma 2005) over a grid of values (strictly greater than 0 and lower than 1, with steps of 0.025 for the scale parameter in RKHS, and steps of 0.05 for the FDR threshold in GBLUP/RKHS-sG). The GCV criterion approximates the leave-one-out cross-validation mean squared error, based on one model fit to the whole training set; it is defined as:

$$GCV=\frac{\left(1/n\right){\left(g-\hat{g}\right)}^{T}\left(g-\hat{g}\right)}{{\left(1-\left(1/n\right)\mathrm{tr}(H)\right)}^{2}}$$,

where tr refers to the trace (the sum of diagonal elements of a matrix); $\hat{g}$, the linear prediction of g, and H, the “hat” (smoothing) matrix, such that $\hat{g}=Hg$, depend on the parameter under tuning.

The GBLUP and RKHS models, as well as their extensions -wG and -sG, were fitted using the R package rrBLUP (Endelman 2011); the BayesA and BayesB models were fitted using the R package BGLR (Pérez and de los Campos 2014); the HEM of Shen *et al.* (2013) was fitted using the R package bigRR (Shen *et al.* 2013), and the RF model was fitted using the R package randomForest (Liaw and Wiener 2002).

#### Marker-data transformations:

As mentioned above, the input X to prediction models were transformations of the marker-data matrix M. For a given set of individuals, consisting of either WS4U-C2, Liberty-C2, or both populations combined, the following transformations of M were made: (i) *Base*, where features are centered allelic dosages, and correspond to the typical input to GS models: $X_{Base}=M-P$, with P the m × q matrix with uniform columns containing the mean allelic dosages within the population, $q=q^{*}$; (ii) *PCA*, where features are uncorrelated synthetic variables each contributed differentially by marker variables: $X_{PCA}=X_{Base}V$, with V the $q^{*}\times d$ rotation matrix in the singular value decomposition of $X_{Base}$ ($X_{Base}=UDV^{T}$; d is the number of principal components here equal to m for all sets of individuals), q=d; (iii) *Cor*, where features are marker variables scaled through a correlation matrix: $X_{Cor}=X_{Base}L$, with L the $q^{*}\times q^{*}$ lower-triangular matrix from the Cholesky decomposition of $\Theta =R^{-1}$, such that $LL^{T}=\Theta$, R being the matrix of correlation between marker variables as previously described, $q=q^{*}$; (iv) *LD*, where features are marker variables weighted based on their relative degree of tagging (the more redundant information at a given marker, the lower its weight): $X_{LD}=X_{Base}W^{1/2}$, with W the diagonal matrix of weights supposed to adjust for redundancy in marker information due to LD; W=diag(w), and w was the least-absolute-error solution to $\left(R\#R\right)w=1_{q^{*}}$ subject to $w_{j}\geq 0$, $j=1,\ldots ,q^{*}$, with R#R the matrix of squared correlation between marker variables and $1_{q^{*}}$ the $q^{*}$-vector of one values, q=16,555 in WS4U-C2, and q=10,615 in Liberty-C2 ($q<q^{*}$, as a result of some weights being exactly zero). The publicly available LDAK software was used to calculate w (http://dougspeed.com/ldak/; Speed *et al.* 2012).

Marker-data transformations were chosen so that they have well-defined equivalencies in the GBLUP and RKHS models. In a GBLUP model, *PCA* is exactly equivalent to *Base* (Figure S1): $\left(X_{Base}V\right){\left(X_{Base}V\right)}^{T}=UDV^{T}VV^{T}VDU^{T}=UD^{2}U^{T}$, since $V^{T}V=I$, and$\;UD^{2}U^{T}$ is the eigendecomposition of $X_{Base}X_{Base}{}^{T}$; *Cor* is equivalent to *Base* when local LD is accounted for through Θ: $\left(X_{Base}L\right){\left(X_{Base}L\right)}^{T}=X_{Base}LL^{T}X_{Base}{}^{T}=X_{Base}\Theta X_{Base}{}^{T}$; *LD* is equivalent to *Base* when local LD is accounted for by weights on marker features as in Speed *et al.* (2012): $\left(X_{Base}W^{1/2}\right){\left(X_{Base}W^{1/2}\right)}^{T}=X_{Base}WX_{Base}{}^{T}$. The equivalencies mentioned for cross-products in GBLUP also apply to Euclidean distances in RKHS. Interestingly, in a RR-BLUP model (equivalent to GBLUP), *Cor* and *LD* correspond to *Base* when marker-feature effects are assumed to follow a $\text{Normal}(0,\Theta \sigma_{\beta }^{2})$, and a $\text{Normal}(0,W\sigma_{\beta }^{2})$, respectively, instead of a $\text{Normal}(0,I\sigma_{\beta }^{2})$ ($\sigma_{\beta }^{2}$ is the variance of marker effects). For the more complex models accounting for heteroscedasticity (BayesA, BayesB, GBLUP-wG, GBLUP-sG, RKHS-wG, RKHS-sG, and RF), the transformed marker variables were considered features in their own right, whose contribution to a given outcome of interest may be weighted similarly to features in *Base*.

Throughout the article, prediction procedures are referred to by a combination of marker-data transformation and prediction model (*e.g.*, *Cor* – RKHS-sG) for a given learning scheme (involving the grouping of populations and environments in a training set; see next subsections).

Matrices X for WS4U-C2 and Liberty-C2 (with transformations *Base*, *PCA*, *Cor*, and *LD*), and the corresponding relationship matrices and Euclidean distance matrices, are available online from http://dfrc.wisc.edu/sniper/, in .rds format readable in R.

#### Population learning schemes:

Given a target population, we considered two types of training sets with regard to parent genotypes. The HS-family BLUPs used for training prediction models could be either from the target population only (within-population learning), or from both populations pooled together (across-population learning).

#### Environment learning schemes:

Given a target outcome, we considered two types of training sets with regard to observations at each parent genotype. For example, with DMY in WI as the target outcome, the HS-family BLUPs used for training the prediction model could be either those from the target location only (within-environment learning; *e.g.*, data on DMY in WI only), n=m, or from both locations considered jointly for the same trait (across-environment learning; *e.g.*, data on DMY in WI and NE), n=2m. In across-environment learning, whenever leaving out HS families from the dataset for validation (see next section), the data on the same HS families in both environments were used for testing.

### Validation of prediction procedures

Prediction procedures were evaluated using prediction accuracy estimated in five-fold cross-validation. Given a random partition of instances in five subsets of similar size, four subsets were used for training, and the remaining subset was used for testing. For each of the five subsets used sequentially for testing, prediction accuracy was computed as the Pearson coefficient of correlation between “observed” and predicted HS-family BLUPs. The significance of the difference in prediction accuracy between a given procedure and a standard procedure was assessed in replicated cross-validation by two-sided paired Dunnett tests, which are *t*-tests modified to account for multiple comparisons to a single control (Dunnett 1964). For each outcome and population, the standard (control) procedure was chosen to be *Base* − GBLUP with within-population and within-environment learning. In cross-validation, the overlap between training sets results in lower variability among estimates of prediction accuracy, compared to the hypothetical case where training sets are generated independently. So, in paired Dunnett tests, the *t*-statistic T was adjusted to account for correlation among computed prediction accuracies, as described in Bouckaert and Frank (2004):

T=Δ¯SD(Δ)1KR+1K−1,

where K=5 is the number of “folds” in cross-validation, and R=10 is the number of replications (five-fold cross-validation was repeated 10 times); $\Delta =z\left(c_{t}\right)-z\left(c_{0}\right)$, with $c_{t}$ and $c_{0}$ the KR-vectors of prediction accuracies from the test procedure and the standard procedure, respectively, and z the Fisher transformation (for normality of prediction accuracies); $\bar{\Delta }$ and SD(Δ) are the mean and SD of Δ, respectively. The R package nCDunnett was used to obtain adjusted p-values for T.

In order to limit the number of possible combinations to assess, prediction procedures were first optimized with respect to learning schemes only, based on replicated cross-validation, and then optimized with respect to marker-data transformation and prediction model in a two-step process (intended to reduce computational burden in the optimization): for each outcome and population, the combination of marker-data transformation and prediction model with the highest prediction accuracy based on nonreplicated cross-validation was selected; then the selected procedure was compared to the standard procedure (*Base* − GBLUP) in replicated cross-validation. If the selected procedure differed from *Base* − GBLUP by both an alternate marker-data transformation and an alternate prediction model (*e.g.*, *Cor* − BayesA), the alternate transformation was first compared to *Base* in a GBLUP model to assess the usefulness of transforming the marker data (*e.g.*, *Cor* − GBLUP *vs.* *Base* − GBLUP). Then the alternate model was compared to GBLUP using the alternate transformation for both models to assess the benefit from a more complex prediction model (*e.g.*, *Cor* − BayesA *vs.* *Cor* − GBLUP). Dunnett tests, with adjustments for the number of marker-data transformations or prediction models, were used in these comparisons in order to account for selection bias, *i.e.*, the fact that the same data were used for both choosing the selected procedure and then comparing it with *Base* − GBLUP.

### Genetic analysis of phenotypic traits

#### Genomic correlation:

To characterize the correlation between genomic effects (u) at different outcomes, a multivariate GBLUP model was fitted on any pair of outcomes as in Burgueño *et al.* (2012): errors e were assumed independent within outcomes; the genomic relationship matrix was $K\propto X_{Base}X_{Base}{}^{T}$; the covariances by outcome of genomic effects and errors were estimated by REML. The R package ASREML-R was used to fit the multivariate GBLUP models.

#### Association mapping:

In order to assess the plausibility of heteroscedastic models, we conducted genome-wide association studies (GWAS) on all outcomes for both populations combined. For each marker in M having a minor allele frequency (MAF) higher than 0.05, the EMMAX linear mixed model of Kang *et al.* (2010), in which relatedness was accounted for through $K\propto X_{Base}X_{Base}{}^{T}$, was fitted using the R package rrBLUP. The threshold used to declare significance of associations was a FDR (as from Storey and Tibshirani 2003) lower than 0.05. Significant markers were then selected altogether in one linear mixed model, with relatedness accounted for through K and fixed effects for markers, using a forward stepwise selection procedure based on the Bayesian information criterion. No covariate for population structure was included in the GWAS models, since the incentive for performing GWAS here was to investigate whether the ability of GBLUP to capture variation at outcomes could be significantly improved by including fixed marker effects. Even though between-population variability was adjusted for—because HS-family BLUPs were computed within each population separately—and genetic relatedness was captured through K in GWAS models, it cannot be ruled out that significant markers actually reflected some population structure.

#### Partition of genomic heritability:

The genomic heritability is defined here as the proportion of variance explained by a GBLUP model, *i.e.*, $\frac{\sigma_{u}^{2}}{\sigma_{u}^{2}+\sigma_{e}^{2}}$. To assess the relative contribution of markers with different degrees of tagging to the genomic heritability, a multiple-component GBLUP model was fitted on each outcome, as in Yang *et al.* (2011), with three nonoverlapping marker classes: genomic relationship matrices $K_{1}$, $K_{2}$, and $K_{3}$ were calculated from the distinct marker classes in $X_{Base}$, and the associated variances $\sigma_{u_{1}}^{2}$, $\sigma_{u_{2}}^{2}$, and $\sigma_{u_{3}}^{2}$ were estimated by REML. The contribution of class ***j*** to the genomic heritability was defined as $\frac{\sigma_{u_{j}}^{2}}{\sum_{j^{’}}\sigma_{u_{j^{’}}}^{2}+\sigma_{e}^{2}}$. Marker classes were determined from tertiles on the markers’ degree of tagging. The R package ASREML-R was used to fit the multiple-component GBLUP models.

### Data availability

The authors state that all data necessary for confirming the conclusions presented in the article are represented fully within the article.

## Results

### Genomic structure and relatedness in populations

**Figure 1:**
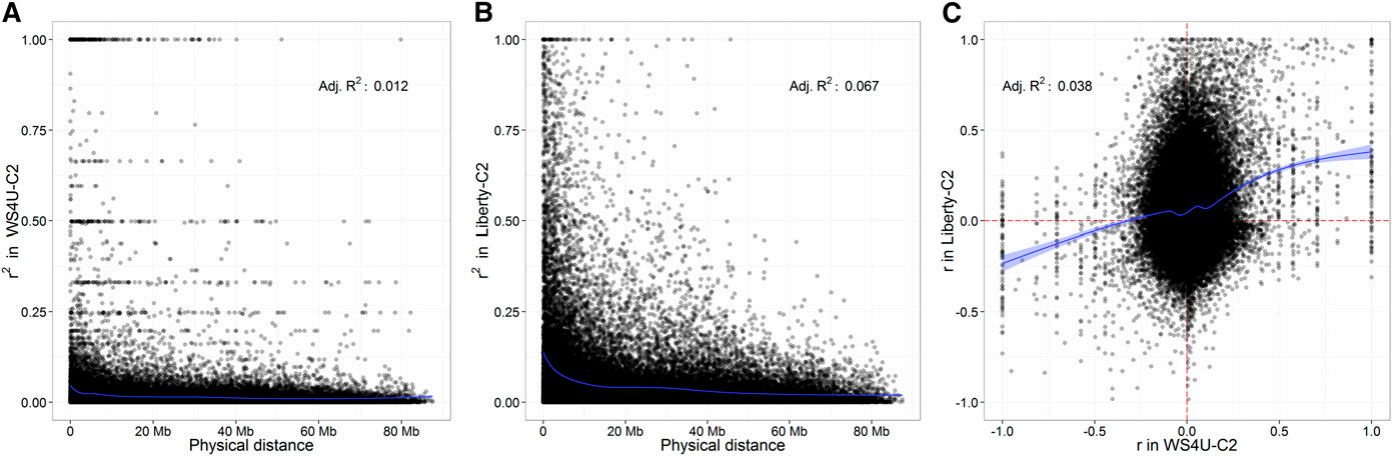
Patterns of decay in linkage disequilibrium (LD), represented by the squared correlation between expected gametic phases (*r*^2^) in (A) WS4U-C2 and (B) Liberty-C2; the blue curve corresponds to the mean value from a cubic-regression spline model assuming a Gamma distribution for *r*^2^. (C) Concordance, from WS4U-C2 to Liberty-C2, in LD as represented by the correlation between expected gametic phases (*r*); the blue curve corresponds to the mean value (and its 95%-confidence interval) from a cubic-regression spline model assuming a Normal distribution for *r* in Liberty-C2. Values of *r* were inferred as described in Weir (1979) using the R package SNPRelate (Zheng *et al.* 2012). Cubic-regression spline models were fitted using the R package mgcv (Wood 2006). The values of *r* and *r*^2^ shown here are based on random pairs of markers polymorphic in both WS4U-C2 and Liberty-C2, with each marker represented only once across all pairs.

The two populations considered, WS4U-C2 and Liberty-C2, had strong differences in their patterns of decay in local (within-chromosome) LD. WS4U-C2 had a rapid decline in local LD, with the squared correlation between expected gametic phases (*r*^2^) being essentially zero for physical distances between markers above 1 Mb (Figure 1A). Conversely, in Liberty-C2, values of *r*^2^ decayed more slowly (Figure 1B), in accordance with the fact that effective population size in this population, derived from a cross between two cultivars, is certainly lower than in WS4U-C2, derived from a diverse collection of 162 upland-ecotype plants. The concordance in LD from WS4U-C2 to Liberty-C2 was very low, as reflected by the low adjusted *R*^2^ in a nonlinear regression for (y = r in Liberty-C2; x = r in WS4U-C2) on *r* in WS4U-C2. This low concordance seemed to be due to the rapid LD decay in WS4U-C2, with many values of *r* being close to zero in WS4U-C2 only (Figure 1C). However, when values of *r* departed from zero in both populations, there seemed to be some consistency in LD phase, as reflected by the sign of *r* being relatively consistent from one population to another.

The distribution of minor allele frequency (MAF), over all 141,030 markers selected, differed by population. In WS4U-C2, as MAF decreased, the cognate number of markers increased exponentially (Figure S2A). In Liberty-C2, while a high proportion of markers appeared to be fixed, or singletons (likely due to *de novo* mutations, or possibly genotype miscalls), the remaining markers showed a relatively uniform distribution in their MAF (Figure S2B). The concordance in MAF from WS4U-C2 to Liberty-C2 was low (adjusted *R*^2^ = 0.24) but significant (p < 0.0001), with many markers being fixed in one population but not the other (Figure S2C).

**Figure 2:**
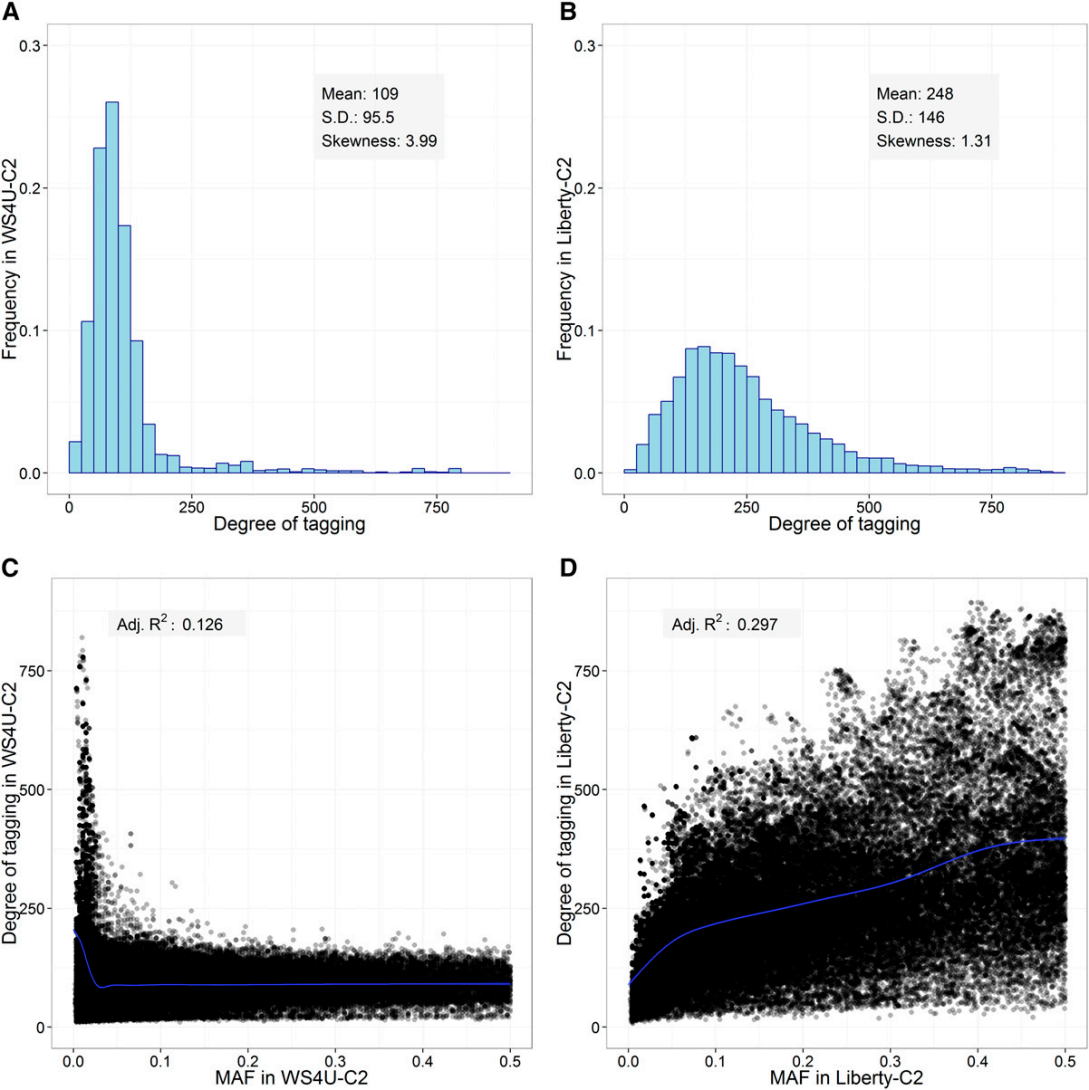
Distribution of degree of tagging in (A) WS4U-C2 and (B) Liberty-C2. Relationship between minor allele frequency (MAF) and degree of tagging in (C) WS4U-C2 and (D) Liberty-C2; the blue curve corresponds to the mean value (and its 95%-confidence interval) from a cubic-regression spline model assuming a Normal distribution for the degree of tagging. Cubic-regression spline models were fitted using the R package mgcv (Wood 2006).

Figure 2, A and B, shows the distribution in the degree of tagging, reflecting the duplication of marker signals along a given chromosome, in each population. The relative variability and skewness in degree of tagging were larger in WS4U-C2 than in Liberty-C2 (respectively: coefficient of variation of 0.876 and 0.591; skewness of 3.99 and 1.31). In Liberty-C2, the relationship between MAF and degree of tagging was typical, with rare variants generally having a low degree of tagging (Figure 2D). Conversely, in WS4U-C2, degree of tagging was, on average, much higher for rare variants (MAF < 0.10) than for more common variants (MAF > 0.10) (Figure 2C). The high degree of tagging for rare variants in WS4U-C2 was due to extended local LD, observed in the LD-decay plots as lines of points for WS4U-C2 (Figure 1A) but not for Liberty-C2 (Figure 1B). We hypothesize that such regions of extended LD in WS4U-C2 are due to the presence, in WS4U (the original collection of 162 plants), of haplotypes consisting of rare alleles, and limited recombination during the two cycles of selection leading to WS4U-C2.

**Figure 3:**
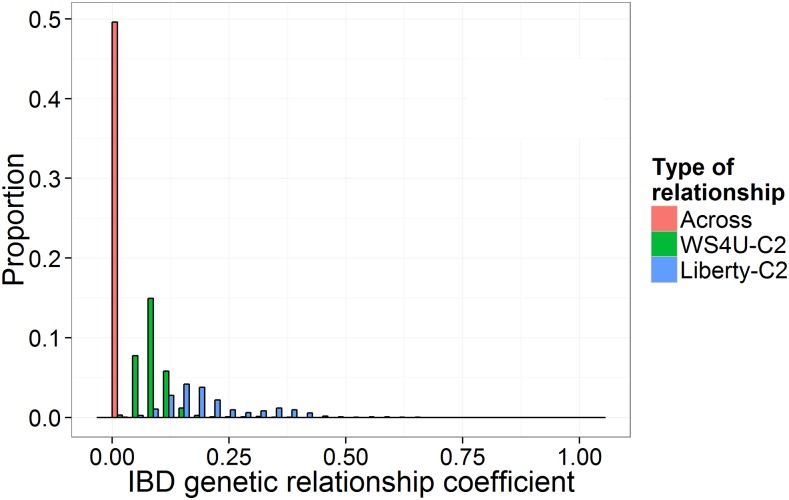
Distribution of genetic relationship coefficients based on identity by descent (IBD) within WS4U-C2, within Liberty-C2, and across populations. Coefficients of IBD were inferred from the EM algorithm described in Milligan (2003) using the R package SNPRelate (Zheng *et al.* 2012); IBD genetic relationship coefficients are equal to twice the IBD coefficients.

Genetic relationship, defined here as twice the coefficient of identity by descent (IBD) between genotypes, was generally lower within WS4U-C2 than within Liberty-C2, in accordance with the presumably higher effective population size in WS4U-C2 (Figure 3). Interestingly, the distribution in genetic relationships within Liberty-C2 was bimodal, with the first peak close to a HS relationship of 1/4, and the second peak close to a full-sib relationship of 1/2. This genetic structure within Liberty-C2 could have been generated by preferential mating of plants from the same ecotype (having similar flowering times), *i.e.*, assortative mating during crosses between the upland and lowland cultivars. Relationships across populations, inferred to be exactly zero, indicated strong genetic dissemblance between WS4U-C2 and Liberty-C2. This dissemblance, along with the low consistency in LD and MAF across populations, suggests little benefit from pooling populations into one single training set for genomic prediction, as was observed in previous studies (*e.g.*, Karoui *et al.* 2012; de los Campos *et al.* 2013).

### Genetic variability and architecture of phenotypic traits

The outcomes considered for genomic prediction, described in Table 1, consisted of combinations of location and phenotypic trait, measured in 2 or 3 yr. Mean reliabilities (*i.e.*, the average inferred squared correlation between true HS-family effects $g_{i}$’s and their BLUPs) were high (≥ 0.61) for PH and HD in both locations. Reliabilities for DMY were relatively high in NE (0.45 in WS4U-C2, and 0.53 in Liberty-C2), but low in WI (0.10 in WS4U-C2, and 0.21 in Liberty-C2).

Genomic correlations between outcomes of the same trait in different environments (based on multivariate GBLUP models) were positive, strong (≥ 0.81), and significant (p ≤ 0.05), with PH and HD (Figure S3), suggesting some benefit from pooling data from different locations into one single training set, for genomic prediction on these two traits. Conversely, genomic correlations between outcomes were not significant (p > 0.05) with DMY, for both populations. Importantly, there were significant genomic correlations between different traits, such as between DMY-NE and PH-NE in WS4U-C2, and between DMY-NE and PH-WI or HD-NE in Liberty-C2. Unfortunately, due to the failure of multiple-trait models to effectively fit the data in cross-validation (likely due to our relatively small sample sizes), no multivariate prediction procedures were considered in this study.

Association analyses were performed on each of the 12 outcomes, for both populations combined. With DMY in WI, seven markers were significantly associated to the outcome, and were selected together in one linear mixed model that explained 42% of the variance after accounting for genetic relatedness, as was reflected by a likelihood-ratio based *R*^2^ (*R*^2^_LR_) of 0.42 (Sun *et al.* 2010) (Table S1). The relatively high *R*^2^_LR_ suggests that DMY in WI may be explained by few markers with strong effects; heteroscedastic models may then be beneficial in genomic prediction with this particular outcome. Conversely, with all other outcomes, no marker was deemed significant, and therefore the corresponding genetic architecture presumably consisted of rare and/or small-effect causal variants.

**Figure 4:**
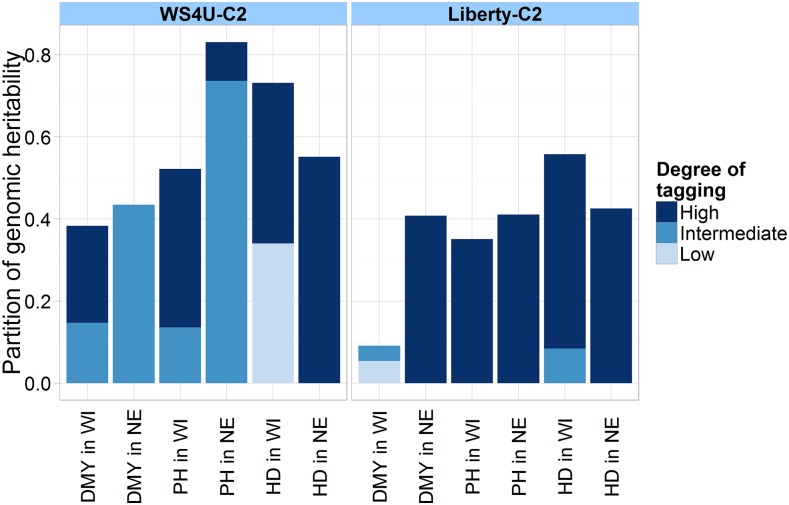
Partition of genomic heritability by marker class based on degree of tagging. “Low”: markers with a degree of tagging lower than the 1/3-quantile; “Intermediate”: markers with a degree of tagging between the 1/3- and 2/3-quantiles; “High”: markers with a degree of tagging higher than the 2/3-quantile. For a given outcome and a given population, the height of the bar corresponding to class *j* indicates its contribution to the total genomic heritability, *i.e.*, $\frac{\sigma_{u_{j}}^{2}}{\sum_{j^{’}}\sigma_{u_{j^{’}}}^{2}+\sigma_{e}^{2}}$, estimated in a multiple-component GBLUP model, with $X_{Base}$ as input, using ASREML-R (Butler *et al.* 2007).

For a given population, the relative contribution of different marker classes to the genomic variance at a given outcome was characterized through the partition of genomic heritability, from a multiple-component GBLUP model. The classes of markers were determined based on the degree of tagging. They consisted of markers with a degree of tagging below the 1/3-quantile, between the 1/3- and 2/3-quantiles, and above the 2/3-quantile, respectively. Quite remarkably, strongly tagged markers seemed to capture a large proportion of the genomic heritability in Liberty-C2 for all outcomes (≥ 85% of the total genomic heritability explained) except DMY in WI, for which the total genomic heritability was low, and the contribution from strongly tagged markers was estimated to be null (Figure 4). In WS4U-C2, the estimated contribution of strongly tagged markers to the total genomic heritability was moderate-to-large (≥ 54%) for DMY in WI, PH in WI, and HD in both locations, but low for PH in NE (11%), and null for DMY in NE (Figure 4). With HD in WI for WS4U-C2 and with DMY in WI for Liberty-C2, weakly tagged markers seemed to capture about half of the total genomic heritability, but, for all other outcomes across populations, weakly tagged markers seemed to capture very little of the genomic heritability.

### Selection and validation of prediction procedures

Here, four components of prediction procedures for GS, on only one outcome at a time for each population, were assayed: (i) population learning scheme—training set consisting of data on either the target population only, or both populations; (ii) environment learning scheme—training set consisting of data on either the target trait-location only, or the same trait in both locations; (iii) marker-data transformation—type of marker features used as input to prediction models; and (iv) prediction model—assumed relationship between the marker features and the outcome.

#### Learning schemes:

The importance of the first two components of prediction procedures (learning schemes) was assessed while using a GBLUP model with *Base* marker-data transformation (*Base* − GBLUP), *i.e.*, using centered expected allelic dosages as input to the standard GBLUP model (Table 2 for DMY and Table S2 for other traits). Across all outcomes, there was no consistent superiority of one particular learning scheme, based on mean prediction accuracies in five-fold cross-validation replicated 10 times. Also, none of the observed increases in prediction accuracy, compared to the within-population/within-environment scheme, were deemed significant (p > 0.10). Pooling data across populations was not beneficial, as could be expected from the low concordance in LD patterns (Figure 1C) and MAF (Figure S2C) between WS4U-C2 and Liberty-C2, as well as from the genetic dissemblance between the two populations (Figure 3). However, no significant increase in prediction accuracy was obtained with PH and HD from pooling data across environments in spite of the strong positive genomic correlations inferred for these two traits (Figure S3).

**Table 2:** Mean prediction accuracy across population and environment learning schemes for DMY in WI and NE

		DMY in WI		DMY in NE	
		Within environment	Across environments	Within environment	Across environments
WS4U-C2	Within population	0.156	0.149	0.136	0.155
	Across populations	0.177	0.158	0.121	0.143
Liberty-C2	Within population	0.095	−0.031	0.495	0.484
	Across populations	0.093	−0.029	0.499	0.485

Prediction accuracies were estimated with Base − GBLUP in five-fold cross-validation replicated 10 times. The significance of differences in prediction accuracy was assessed by two-sided paired Dunnett tests, which accounted for multiple comparisons of learning schemes to a single reference (the within-population/within-environment scheme). The *t*-statistics in Dunnett tests were adjusted to account for correlation among training sets in cross-validation, as described in Bouckaert and Frank (2004). For a given population and trait-location combination, differences in prediction accuracy compared to the within-population/within-environment scheme were never deemed significant (p > 0.10 in paired Dunnett tests).

#### Marker-data transformations and prediction models:

**Figure 5:**
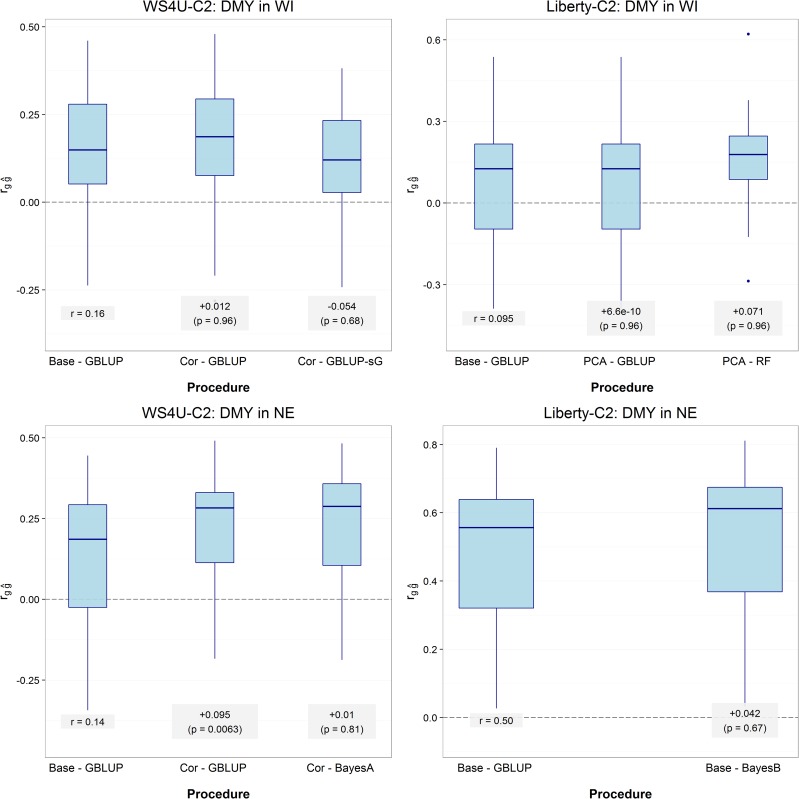
Validation of selected prediction procedures for DMY in WI and NE. Prediction accuracies ($r_{g\hat{g}}$) were estimated with a within-population/within-environment learning scheme in five-fold cross-validation, replicated 10 times. In each boxplot, up to two comparisons are made: (i) the candidate-transformation procedure (selected marker-data transformation according to nonreplicated five-fold cross-validation in a GBLUP model; Table 3) is compared to the standard procedure (Base − GBLUP), if relevant; and (ii) the candidate procedure (selected prediction procedure according to nonreplicated five-fold cross-validation; Table 3) is compared to the candidate-transformation procedure. The significance of differences in prediction accuracies was assessed by two-sided paired Dunnett tests, which accounted for multiple testing of data transformations, in (i), and of prediction models, in (ii). The *t*-statistics in Dunnett tests were adjusted to account for correlation among training sets in cross-validation, as described in Bouckaert and Frank (2004).

As described above, pooling data across populations and/or environments did not offer strong opportunities for improving prediction procedures. Thus, the importance of marker-data transformations and prediction models was investigated while using a simple within-population/within-environment learning scheme. Assessment of prediction procedures was performed in two steps. In step 1, a candidate prediction procedure (a combination of marker-data transformation and prediction model) was selected based on mean prediction accuracy in nonreplicated five-fold cross-validation (Table 3 for DMY, Table S3 for other traits, and Figure S4). In step 2, the selected candidate procedure was then compared to the standard—a GBLUP model on centered expected allelic dosages (*Base* − GBLUP)—in five-fold cross-validation replicated 10 times (Figure 5 for DMY, Figure S5 for other traits) so as to assess the benefit from the alternate marker-data transformation, and/or the more complex prediction model selected.

**Table 3:** Mean prediction accuracy across marker-data transformations and prediction models for DMY in WI and NE

		DMY in WI									
		GBLUP	GBLUP-wG	GBLUP-sG	RKHS	RKHS-wG	RKHS-sG	BayesA	BayesB	RF	(Mean)
WS4U-C2	*Base*	0.151	0.123	0.106	0.139	0.135	0.114	0.135	0.121	0.037	0.118
	*PCA*	0.151	−0.045	−0.010	0.139	0.027	−0.017	0.102	0.082	0.105	0.059
	*Cor*	0.170	0.152	**0.200**	0.146	0.155	0.194	0.150	0.122	0.120	0.157
	*LD*	0.105	0.107	0.092	0.113	0.105	0.070	0.105	0.104	0.031	0.092
	(Mean)	0.144	0.084	0.097	0.134	0.106	0.090	0.123	0.107	0.073	0.106
Liberty-C2	*Base*	0.016	−0.019	0.069	−0.038	0.003	0.064	−0.046	−0.079	−0.008	−0.004
	*PCA*	0.016	0.027	0.092	−0.038	0.081	0.083	−0.021	−0.028	**0.165**	0.042
	*Cor*	0.004	−0.054	−0.045	−0.211	−0.062	−0.037	−0.061	−0.093	−0.115	−0.075
	*LD*	0.034	−0.044	0.015	−0.053	−0.028	0.031	−0.017	−0.039	0.018	−0.009
	(Mean)	0.018	−0.023	0.033	−0.085	−0.002	0.035	−0.036	−0.06	0.015	−0.012

		DMY in NE									
		GBLUP	GBLUP-wG	GBLUP-sG	RKHS	RKHS-wG	RKHS-sG	BayesA	BayesB	RF	(Mean)
WS4U-C2	*Base*	0.079	0.053	0.096	0.061	0.064	0.122	0.096	0.068	0.132	0.086
	*PCA*	0.079	−0.048	−0.040	0.061	−0.014	−0.002	0.079	0.081	0.039	0.026
	*Cor*	0.174	0.145	0.168	0.162	0.149	0.153	**0.185**	0.165	0.031	0.148
	*LD*	0.151	0.172	0.155	0.129	0.159	0.148	0.159	0.149	0.075	0.144
	(Mean)	0.121	0.081	0.095	0.103	0.090	0.105	0.130	0.116	0.069	0.101
Liberty-C2	*Base*	0.493	0.468	0.444	0.498	0.474	0.444	0.520	**0.526**	0.439	0.478
	*PCA*	0.493	0.313	0.275	0.498	0.379	0.343	0.505	0.495	0.419	0.413
	*Cor*	0.479	0.468	0.440	0.455	0.470	0.443	0.493	0.503	0.456	0.467
	*LD*	0.474	0.462	0.408	0.481	0.474	0.409	0.511	0.495	0.414	0.459
	(Mean)	0.485	0.428	0.392	0.483	0.449	0.41	0.507	0.505	0.432	0.455

Prediction accuracies were estimated with a within-population/within-environment learning scheme in five-fold cross-validation, with no replication. For a given population and outcome (trait-location combination), the highest average value across marker-data transformations is underlined; the highest value across prediction procedures is underlined and bolded.

Over cases (combinations of outcome and population), average prediction accuracies across prediction procedures in nonreplicated cross-validation ranged from –0.012 (DMY in WI for Liberty-C2) to 0.545 (HD in WI for Liberty-C2), with generally higher accuracies with PH and HD than with DMY (Table 3, Table S3, and Figure S4). However, high accuracies were achieved with DMY in NE for Liberty-C2 (0.455 on average). With DMY in WI (for both populations), DMY in NE (for WS4U-C2), and PH in NE (for WS4U-C2), marker-data transformation seemed to offer more opportunities for improvement than prediction models. In other cases, prediction accuracies were generally not sensitive to marker-data transformations, except for *PCA*, with which heteroscedastic models often performed poorly. In general, across cases, the *Base* marker-data transformation was selected in only three out of 12 cases, which again indicates some potential benefit from accounting for LD through marker-data transformation. Heteroscedastic models were selected nine times out of 12, with strong increases observed with DMY in WI, as could be expected from the many GWAS signals detected with that trait (Table S1).

The statistical significance of the difference in prediction accuracies between *Base* − GBLUP and the candidate prediction procedure, selected based on nonreplicated cross-validation, was assessed in replicated cross-validation by paired Dunnett tests. Dunnett tests account for biases on significance due to multiple testing, which arose here from deriving a hypothesis from the same dataset as that used for testing (selection bias). Dunnett tests were further adjusted to account for the overlap between training sets in cross-validation, which caused the SD in prediction accuracy among “folds” to be an underestimate of the general SD (among hypothetical datasets). With DMY and PH in NE for WS4U-C2, and with DMY (in both locations) for Liberty-C2, the selected prediction procedures yielded higher mean prediction accuracies in replicated cross-validation. However, the observed differences were generally not deemed significant according to our tests, with the exception of DMY in NE for WS4U-C2 (Figure 5 and Figure S5). With DMY and PH in NE for WS4U-C2, the marker-data transformation apparently contributed more than the prediction model to the increase in prediction accuracy. Interestingly, for those cases, markers with a high degree of tagging tended to capture very little of the genomic heritability (Figure 4). The most notable case was DMY in NE for WS4U-C2, where a highly significant increase in accuracy (p < 0.01) was observed when comparing *Base* − GBLUP to *Cor* − GBLUP, but using a BayesA model (the selected alternate model) rather than a GBLUP model (the standard model) did not yield a significant increase in prediction accuracy (Figure 5). This one strong increase indicates that, with our data, some substantial and reliable increase in prediction accuracy could be achieved through marker-data transformation, and not so much by prediction models more complex than GBLUP. Accordingly, when going through the same process of selection and validation of prediction procedures while considering only *Base* for input to the various prediction models (which is a traditional protocol for prediction-procedure optimization in GS), no significant increase in prediction accuracy could be obtained in any case (Figure S6).

## Discussion

This study dealt with the optimization of prediction procedures in GS through four components: population learning scheme, environment learning scheme, marker-data transformation, and prediction model, with emphasis placed on marker-data transformations and prediction models. The distinction between marker-data transformations and prediction models may seem arbitrary, especially for procedures like *Cor* − GBLUP or *Cor* − RKHS, which are equivalent to well-defined statistical models (see *Material and Methods*). However, “marker-data transformations” and “prediction models” account for different characteristics of marker loci, with marker-data transformations accounting for redundancy in marker information, and prediction models all assuming independence of marker-feature effects, and possibly accounting for heteroscedasticity and/or nonlinearity of marker-feature effects.

In GS studies, the type of prediction models has been by far the factor upon which optimization of genomic prediction procedures has most often acted (*e.g.*, Moser *et al.* 2009; Crossa *et al.* 2010; Heslot *et al.* 2012). In this study, we demonstrate that applying linear transformations to the marker data to account for local LD among markers may be useful to achieve gains in prediction accuracy that are reliable based on replicated cross-validation and honest significance tests. In fact, the only highly significant increase in prediction accuracy that we achieved was due to *Cor* (the account of local LD among marker loci through a block-diagonal correlation matrix), with DMY in NE for WS4U-C2 (Figure 5). Some substantial increase in prediction accuracy could also be achieved by *Cor* with PH in NE for WS4U-C2, and *PCA* with DMY in WI for Liberty-C2, though the differences observed were not deemed significant (p = 0.37 and p = 0.96, respectively; Figure S5 and Figure 5). While optimizing prediction procedures through learning schemes has been useful in some studies (Rincent *et al.* 2012; Heslot *et al.* 2013), here they did not offer strong opportunities for improving prediction procedures. With PH and HD, for which genomic correlations between environments were high (Figure S3), the absence of consistent and significant increase in prediction accuracy from pooling environments may have been due to the high reliability of HS-family BLUPs for those two traits, causing additional measurements on one given genotype to contribute little to the quality of the signal in the data. Pooling data from different populations was not useful due to the strong genetic dissimilarities between WS4U-C2 and Liberty-C2 (Figure 1, Figure S2, and Figure 3), certainly owing to the differences in effective population sizes, as well as to local adaptation of switchgrass populations and the ancient divergence between upland and lowland ecotypes (Zhang *et al.* 2011).

In our study, both populations showed large variability in the degree of (local) tagging at marker loci (Figure 2, A and B), probably (to some extent) because markers were derived from exome capture, which target specific regions in the genome for sequencing. In WS4U-C2 specifically, there was also rapid LD decay along chromosomes with nevertheless extended LD (Figure 1A), presumably caused by sampling artifacts in WS4U, resulting in outstandingly high values of degree of tagging (Figure 2A). Also, variants with extreme degrees of tagging in that population tended to be rare (Figure 2C). Conversely, in Liberty-C2, LD decay within a chromosome was slower, and there was no extended LD (Figure 1B), resulting in relatively little skewness in the distribution of degree of tagging in that population (Figure 2B). Also, the relationship between MAF and degree of tagging was quite typical (Figure 2D), similar to that reported in human by Speed *et al.* (2012). Probably as a consequence of the differences in genomic structure across populations, the contribution of strongly tagged variants to the genomic heritability of outcomes was generally lower in WS4U-C2 than in Liberty-C2. The only cases in which there were substantial increases in prediction accuracy from marker-data transformations and prediction models, relatively to *Base* − GBLUP, were DMY and PH in NE for WS4U-C2, and DMY in WI for Liberty-C2: +75% from *Cor*-BayesA, +20% from *Cor*-BayesB, and +75% from *PCA*-RF, respectively (Figure 5). Quite remarkably, these were also the only cases in which proportions of genomic heritability explained by strongly tagged variants were very low (0%, 11%, and 0% of genomic heritability explained, in these three cases, respectively) (Figure 4). Importantly, previous simulation studies have investigated the effect of marker-data transformation *LD* on the accuracy of estimations (Speed *et al.* 2012) and predictions (Nishio and Satoh 2015) in genomic studies, and they have shown that the lower the relative degree of tagging at the causal variants for a given outcome, the more beneficial *LD* tends to be, compared to *Base*. Therefore, here we argue that cases where marker-data transformations might be useful are those where a small proportion of genomic heritability is captured by strongly tagged markers, which presumably derives from the fact that the causal variants are not strongly tagged. However, we are assuming here that *Cor* and *PCA* respond to features of genomic structure similarly to *LD*; simulation studies would be necessary to support such an assumption.

In Liberty-C2, there seemed to be some population structure, presumably caused by assortative mating, while there was little evidence for such structure in WS4U-C2 (Figure 3). Population structure may cause marker loci from different chromosomes to be correlated. Therefore, in Liberty-C2 particularly, with marker-data transformations *Cor* and *LD*, it might have been useful to account for global LD, *i.e.*, correlations among marker loci within and across chromosomes. Interestingly, *PCA* does account for global LD in heteroscedastic models (in GBLUP and RKHS, the homoscedastic models, *PCA* is equivalent to *Base*), and this transformation was useful only in Liberty-C2, in one case (DMY in WI). However, despite being substantial, the increase in mean prediction accuracy in this case was not deemed significant in paired comparisons (Figure 5). Here, we did not account for global LD in *Cor* and *LD*, since the transformations would then have involved correlation matrices that are too large to process, or even store. Given the genomic features of Liberty-C2, pruning markers—which should not be too detrimental to prediction accuracy given the relatively large LD extent (Figure 1B)—and then accounting for global LD, might have proved useful with *Cor* and *LD*.

In general, the account of LD through marker-data transformations based on correlation matrices (*Cor* and *LD*) could be further improved by reducing the level of noise in estimates of marker correlations. Preprocessing correlation matrices for shrinkage and/or sparsity could then prove beneficial, with Gaussian graphical models (*e.g.*, the graphical LASSO; Friedman *et al.* 2008) or generalized thresholding methods (*e.g.*, the MCP method; Zhang 2010) being potentially useful tools. Furthermore, basing correlations on expected gametic phases (*i.e.*, using haplotypic rather than genotypic correlations) would allow a more appropriate account of LD. However, in *Cor* (whenever preprocessing correlations or using expected gametic phases), one would then face the very serious computational challenge of ensuring that the resulting correlation matrix is positive definite (*i.e.*, being a proper and invertible correlation matrix). Filtering out markers based on relatively stringent MAF thresholds could result in higher prediction accuracies if the outcome is affected mostly by common causal variants, but it could also increase the benefit from marker-data transformations such as *Cor* or *LD*, because correlations would probably be less prone to error if only common markers are considered—some correlations involving rare variants, estimated at low but nonzero values, might simply be spurious rather than effectively due to LD, and would then contribute to overall noise in the marker correlation matrix. However, preselection based on MAF would remove potentially useful information whereas the linear mixed models assayed here (*i.e.*, all prediction models except RF) have the (supposedly desirable) property of downweighing the effect of markers with low variance (and therefore low MAF) as long as marker variables are not standardized (*i.e.*, scaled through a covariance matrix). Besides, optimizing prediction procedures with respect to a MAF threshold would result in more intricate studies: if optimization for MAF threshold were to be integrated into a prediction procedure (*i.e.*, tuning for MAF threshold within the procedure), the resulting computational complexity would greatly increase, since tuning would then have to be performed within each “fold” during cross-validation for each outcome and population; if different MAF thresholds were to be considered for different procedures (*i.e.*, the MAF threshold would be an additional component of prediction procedures), then the computational burden in the study would increase, but, more importantly, multiple testing would become a greater issue (accounting for the high number of candidates in comparisons of prediction procedures would cause significance tests to be highly conservative). In this study, we considered marker-data transformations that were relatively simple, but some of the treatments suggested above (regularization on correlation matrices, use of expected gametic phases for estimating correlations, and/or preselection based on MAF) may prove useful to better account for LD in GS.

Our results are based on two populations of switchgrass with three traits. The conclusions that we drew about the relative importance of prediction-procedure components are, of course, not generalizable to all GS contexts and genetic architectures. The limited number of genotypes (137 and 110 in WS4U-C2 and Liberty-C2, respectively) likely favored the most parsimonious (statistically efficient) models, regardless of traits’ genetic architecture. With larger sample sizes, the higher flexibility of more complex models may have been more beneficial with traits whose genetic architecture substantially deviates from the infinitesimal model. Also, not all genetic architectures were represented in our data. In particular, there was little apparent benefit from RKHS (the only nonlinear homoscedastic model) on prediction accuracy. This may indicate that the traits considered in our data are influenced mostly by additive effects, but the small sample sizes arguably limit such conclusions. Finally, assessments of GS through cross-validations is limited in that it does not test prediction procedures for persistency of accuracy over generations, a criterion by which prediction models can differ significantly, as was shown through simulations by Habier *et al.* (2007). It would be important to formally study the repercussion of accounting for LD in GS on the persistence of accuracy over generations, using simulation and/or empirical studies. Quite interestingly, Nishio and Satoh (2015) suggested that accounting for LD through the *LD* marker-data transformation would be beneficial in long-term GS when causal variants are unevenly tagged, because it would prevent strongly tagged causal variants from being quickly fixed relatively to weakly tagged ones, and therefore would result in genomic predictions from *LD* − GBLUP having accuracies that are more slowly deteriorated over generations, compared to predictions from *Base* − GBLUP.

We believe the relatively high prediction accuracies, particularly with DMY in NE, should motivate the implementation of GS breeding programs in switchgrass. Nevertheless, we may conduct future studies to compare GS with phenotypic selection for realized genetic gains, in programs that are run in similar conditions, so as to bring further evidence for the usefulness of GS technologies in perennial grass breeding.

## 

## Supplementary Material

Supplemental Material
