# Supplementary material for: Accuracy of Genomic Prediction in Switchgrass (Panicum virgatum L.) Improved by Accounting for Linkage Disequilibrium
Source: G3 (Bethesda). 2016 Feb 10;6(4):1049–62. doi: 10.1534/g3.115.024950 (PMC4825640; doi:10.1534/g3.115.024950)
Supplement: Supplemental Material [file supp_6_4_1049__index.html]

Accuracy of Genomic Prediction in Switchgrass (Panicum virgatum L.) Improved by Accounting for Linkage Disequilibrium — Supplemental Material 

# Accuracy of Genomic Prediction in Switchgrass (*Panicum virgatum* L.) Improved by Accounting for Linkage Disequilibrium

## Supplemental Material for Ramstein *et al.*, 2016

**Files in this Data Supplement:**

- Supplemental Materials - This file contains Supporting Figures and Tables, as well as, legends for Supporting Files. (.pdf, 1,431 KB)
- Figure S1 - Genetic relationship coefficients in WS4U-C2 (upper panel) and Liberty-C2 (lower panel), based on marker features from alternate data transformations (PCA, Cor or LD; see section Material and methods), compared to Base (only centering of expected allelic dosages). (.pdf, 94 KB)
- Figure S2 - Distribution of minor allele frequency (MAF) in (a) WS4U-C2 and (b) Liberty-C2; (c) Concordance of MAF from WS4U-C2 to Liberty-C2; the blue curve corresponds to the mean value (and its 95%-confidence interval) from a cubic-regression spline model assuming a Normal distribution for MAF in Liberty-C2. (.pdf, 382 KB)
- Figure S3 - Heatmaps of genomic correlations between trait BLUPs for traits measured (a) in WS4U-C2; (b) in Liberty-C2. (.pdf, 194 KB)
- Figure S4 - Bar plots of mean prediction accuracies from non-replicated five-fold cross-validation for all outcomes. (.pdf, 248 KB)
- Figure S5 - Validation of selected prediction procedures for PH and HD in WI and NE. (.pdf, 349 KB)
- Figure S6 - Validation of selected prediction procedures for all outcomes, ignoring all alternate marker-data transformations (i.e., other than Base). (.pdf, 388 KB)
- Table S1 - Results of association mapping for DMY in WI across both populations. (.pdf, 119 KB)
- Table S2 - Mean prediction accuracy across population and environment learning schemes for PH and HD in WI and NE. (.pdf, 85 KB)
- Table S3 - Mean prediction accuracy across marker-data transformations and statistical models for PH and HD in WI and NE. (.pdf, 46 KB)
- File S1 - Raw phenotypic data. (.xls, 3,849 KB)
- File S2 - Half-sib (HS) family BLUPs. (.xls, 64 KB)
- File S3 - Untransformed genotype calls at marker loci. (.rar, 9,293 KB)
