# Supplementary material for: Accuracy of Genomic Prediction in Switchgrass (Panicum virgatum L.) Improved by Accounting for Linkage Disequilibrium
Source: G3 (Bethesda). 2016 Feb 10;6(4):1049–62. doi: 10.1534/g3.115.024950 (PMC4825640; doi:10.1534/g3.115.024950)
Supplement: Supplemental Material [file supp_g3.115.024950_024950SM.pdf]

Table S1 – *Results of association mapping for DMY in WI across both populations*

Gene	Feature	Chromosome	Position (bp)	MAF	R^2_{LR}
**Pavir.Ba03876**	intronic	Chr02a	77,527,751	0.097	0.42
**Pavir.Ga00641**	exonic	Chr07a	8,145,980	0.085	
**Pavir.Ha00300**	intronic	Chr08a	6,097,945	0.091	
**Pavir.Ia03558**	intronic	Chr09a	69,972,787	0.081	
**Pavir.Bb00675**	exonic	Chr02b	11,682,163	0.119	
**Pavir.Bb01690**	intronic	Chr02b	41,239,077	0.139	
**Pavir.Hb00247**	exonic	Chr08b	4,667,943	0.053	

Gene names and marker localizations are based on the reference genome sequencing and annotation of Panicum virgatum v1.1 (DOE-JGI, http://phytozome.jgi.doe.gov/)*. R^2_{LR}: likelihood-ratio-based R^2 statistic (Sun et al. 2010). Association mapping was performed using a linear mixed model with the outcome being DMY, PH or HD in WI or NE, with one fixed additive effect for a given marker assayed and a random background effect accounted for through a realized genetic relationship matrix derived from marker data as $\mathbf{K} \propto \mathbf{X}_{Base}\mathbf{X}_{Base}^{\mathrm{T}}$. The threshold used to declare significance of associations was a false discovery rate (as from Storey and Tibshirani 2003) lower than 0.05. The significant markers reported above were selected altogether in one linear mixed model, with a random background effect and fixed effects for markers, by forward stepwise selection based on the Bayesian information criterion. Significant associations were obtained only with DMY in WI (not with the eleven other outcomes).*

Table S2 - Mean prediction accuracy across population and environment learning schemes for PH and HD in WI and NE

		PH in WI		PH in NE	
		Within env.	Across env.	Within env.	Across env.
**WS4U-C2**	Within pop.	0.254	0.253	0.292	0.297
	Across pop.	0.235	0.247	0.289	0.297
**Liberty-C2**	Within pop.	0.329	0.364	0.460	0.458
	Across pop.	0.334	0.367	0.461	0.454
		**HD in WI**		**HD in NE**	
		Within env.	Across env.	Within env.	Across env.
**WS4U-C2**	Within pop.	0.312	0.314	0.268	0.285
	Across pop.	0.291	0.300	0.272	0.285
**Liberty-C2**	Within pop.	0.530	0.519	0.421	0.448
	Across pop.	0.506	0.478	0.410	0.431

Prediction accuracies were estimated with Base – GBLUP in five-fold cross-validation replicated ten times. The significance of differences in prediction accuracy was assessed by two-sided paired Dunnett tests, which accounted for multiple comparisons of learning schemes to a single reference (the within-population/within-environment scheme). The t-statistics in Dunnett tests were adjusted to account for correlation among training sets in cross-validation, as described in Bouckaert and Frank (2004). For a given population and trait-location combination, differences in prediction accuracy compared to the within- population/within-environment scheme were never deemed significant ($p > 0.10$ in paired Dunnett tests).

Table S3 - Mean prediction accuracy across marker-data transformations and statistical models for PH and HD in WI and NE.

PH in WI

		GBLUP	GBLUP-wG	GBLUP-sG	RKHS	RKHS-wG	RKHS-sG	BayesA	BayesB	RF	(Mean)
**WS4U-C2**	**Base**	0.298	0.291	0.238	0.303	0.291	0.213	0.300	0.302	0.086	0.258
	**PCA**	0.298	0.082	0.040	0.303	0.106	0.078	0.196	0.170	0.194	0.163
	**Cor**	0.289	0.259	0.153	0.279	0.263	0.205	0.292	0.306	**0.313**	0.262
	**LD**	0.288	0.302	0.196	0.293	0.291	0.212	0.286	0.294	0.064	0.247
	**(Mean)**	0.293	0.234	0.157	0.2945	0.238	0.177	0.269	0.268	0.164	0.233
**Liberty-C2**	**Base**	0.344	0.315	0.236	0.319	0.299	0.136	0.322	0.315	0.132	0.269
	**PCA**	0.344	0.195	0.085	0.319	0.24	0.141	0.328	0.344	-0.046	0.217
	**Cor**	0.299	0.249	0.225	0.282	0.253	0.203	0.277	0.267	0.270	0.258
	**LD**	**0.371**	0.320	0.196	0.348	0.315	0.187	0.361	0.37	0.173	0.293
	**(Mean)**	0.340	0.270	0.186	0.317	0.277	0.167	0.322	0.324	0.132	0.259

PH in NE

		GBLUP	GBLUP-wG	GBLUP-sG	RKHS	RKHS-wG	RKHS-sG	BayesA	BayesB	RF	(Mean)
**WS4U-C2**	**Base**	0.326	0.312	0.321	0.326	0.320	0.328	0.320	0.326	0.127	0.301
	**PCA**	0.326	0.130	0.130	0.326	0.159	0.159	0.198	0.188	0.187	0.200
	**Cor**	0.372	0.356	0.360	0.374	0.365	0.385	0.386	**0.396**	0.288	0.365
	**LD**	0.290	0.280	0.227	0.309	0.295	0.269	0.291	0.291	0.003	0.251
	**(Mean)**	0.329	0.270	0.260	0.334	0.285	0.285	0.299	0.300	0.151	0.279
**Liberty-C2**	**Base**	**0.500**	0.482	0.418	0.469	0.466	0.295	0.500	0.481	0.381	0.444
	**PCA**	0.500	0.377	0.377	0.469	0.398	0.398	0.496	0.499	0.237	0.417
	**Cor**	0.474	0.456	0.410	0.453	0.449	0.417	0.473	0.470	0.379	0.442
	**LD**	0.477	0.464	0.426	0.453	0.452	0.317	0.479	0.472	0.380	0.436
	**(Mean)**	0.488	0.445	0.408	0.461	0.441	0.357	0.487	0.481	0.344	0.435

HD in WI											
		**GBLUP**	**GBLUP-wG**	**GBLUP-sG**	**RKHS**	**RKHS-wG**	**RKHS-sG**	**BayesA**	**BayesB**	**RF**	**(Mean)**
**WS4U-C2**	**Base**	0.254	0.262	0.207	0.271	0.271	0.224	0.271	0.217	0.129	0.234
	**PCA**	0.254	0.070	0.041	0.271	0.114	0.091	0.204	0.179	0.008	0.137
	**Cor**	0.224	0.200	0.101	0.232	0.209	0.094	0.212	0.224	0.157	0.184
	**LD**	0.234	0.247	0.234	0.249	0.257	0.238	**0.280**	0.192	0.035	0.218
	**(Mean)**	0.242	0.195	0.146	0.256	0.213	0.162	0.242	0.203	0.082	0.193
**Liberty-C2**	**Base**	0.577	0.598	0.599	0.581	0.595	0.587	0.565	0.555	0.543	0.578
	**PCA**	0.577	0.446	0.352	0.581	0.509	0.428	0.570	0.569	0.373	0.489
	**Cor**	0.528	0.550	**0.604**	0.527	0.547	0.599	0.528	0.517	0.504	0.545
	**LD**	0.563	0.564	0.583	0.571	0.567	0.579	0.565	0.560	0.557	0.568
	**(Mean)**	0.561	0.540	0.535	0.565	0.555	0.548	0.557	0.550	0.494	0.545
**HD in NE**											
		**GBLUP**	**GBLUP-wG**	**GBLUP-sG**	**RKHS**	**RKHS-wG**	**RKHS-sG**	**BayesA**	**BayesB**	**RF**	**(Mean)**
**WS4U-C2**	**Base**	0.246	0.233	0.230	0.239	0.232	0.240	0.193	0.226	0.051	0.210
	**PCA**	0.246	0.151	0.114	0.239	0.202	0.187	0.230	0.229	0.139	0.193
	**Cor**	0.212	0.179	0.161	0.230	0.197	0.161	0.214	**0.271**	0.269	0.210
	**LD**	0.213	0.158	0.162	0.208	0.166	0.195	0.213	0.216	0.084	0.179
	**(Mean)**	0.229	0.180	0.167	0.229	0.199	0.196	0.213	0.236	0.136	0.198
**Liberty-C2**	**Base**	**0.420**	0.413	0.351	0.396	0.398	0.295	0.406	0.399	0.369	0.383
	**PCA**	0.420	0.298	0.073	0.396	0.330	0.169	0.387	0.366	0.077	0.280
	**Cor**	0.368	0.358	0.331	0.353	0.354	0.345	0.356	0.360	0.321	0.350
	**LD**	0.403	0.397	0.343	0.376	0.383	0.328	0.400	0.409	0.382	0.380
	**(Mean)**	0.403	0.367	0.275	0.380	0.366	0.284	0.387	0.384	0.287	0.348

Prediction accuracies were estimated with a within-population/within-environment learning scheme in five-fold cross-validation, with no replication. For a given population and outcome (trait-location combination), the highest average value across marker-data transformations is underlined; the highest value across prediction procedures is underlined and bolded.

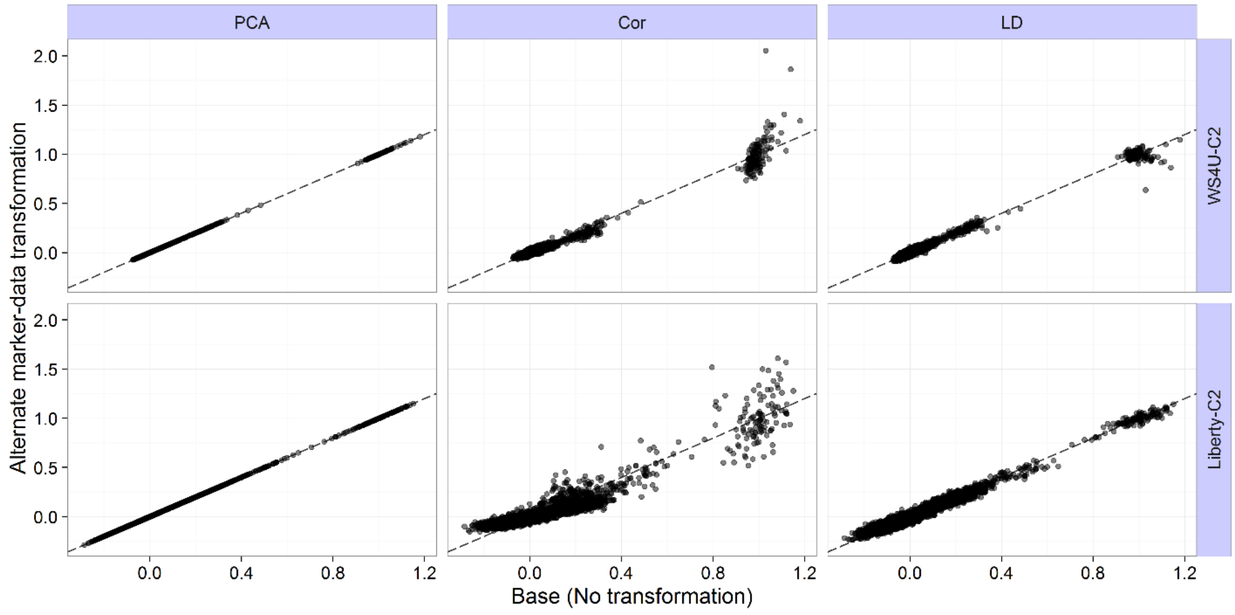

Figure S1 – *Genetic relationship coefficients in WS4U-C2 (upper panel) and Liberty-C2 (lower panel), based on marker features from alternate data transformations (PCA, Cor or LD; see section Material and methods), compared to Base (only centering of expected allelic dosages).*

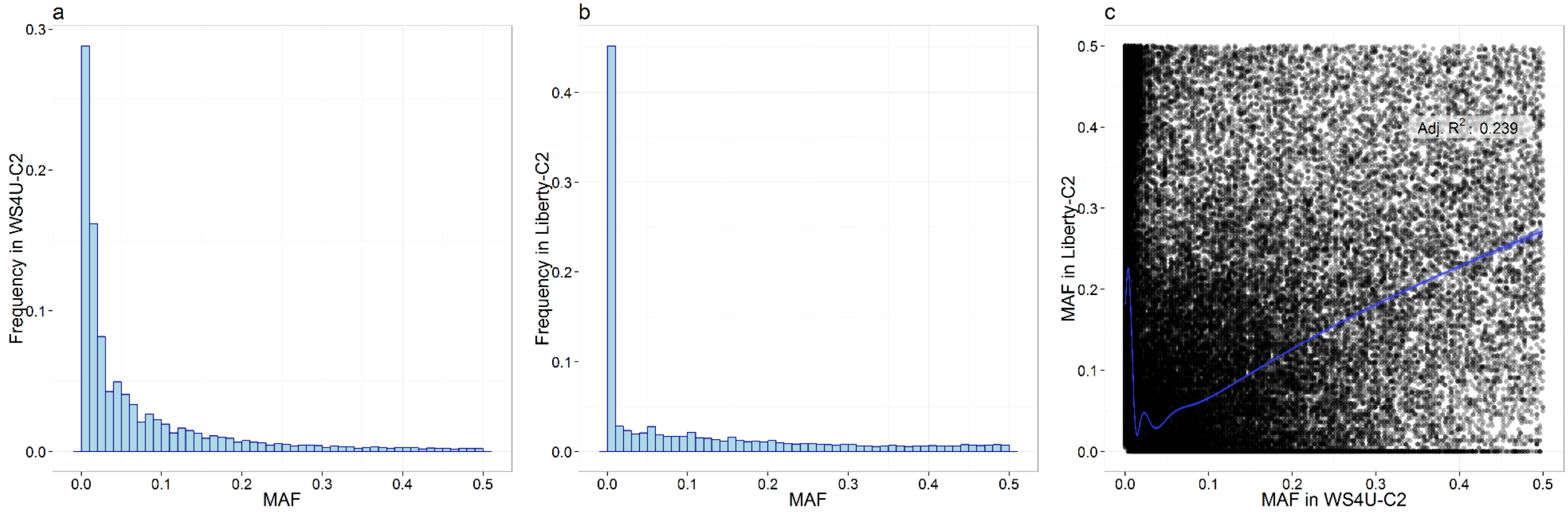

Figure S2 – *Distribution of minor allele frequency (MAF) in (a) WS4U-C2 and (b) Liberty-C2; (c) Concordance of MAF from WS4U-C2 to Liberty-C2; the blue curve corresponds to the mean value (and its 95%-confidence interval) from a cubic-regression spline model assuming a Normal distribution for MAF in Liberty-C2. Cubic-regression spline models were fitted using the R package mgcv (Wood 2006).*

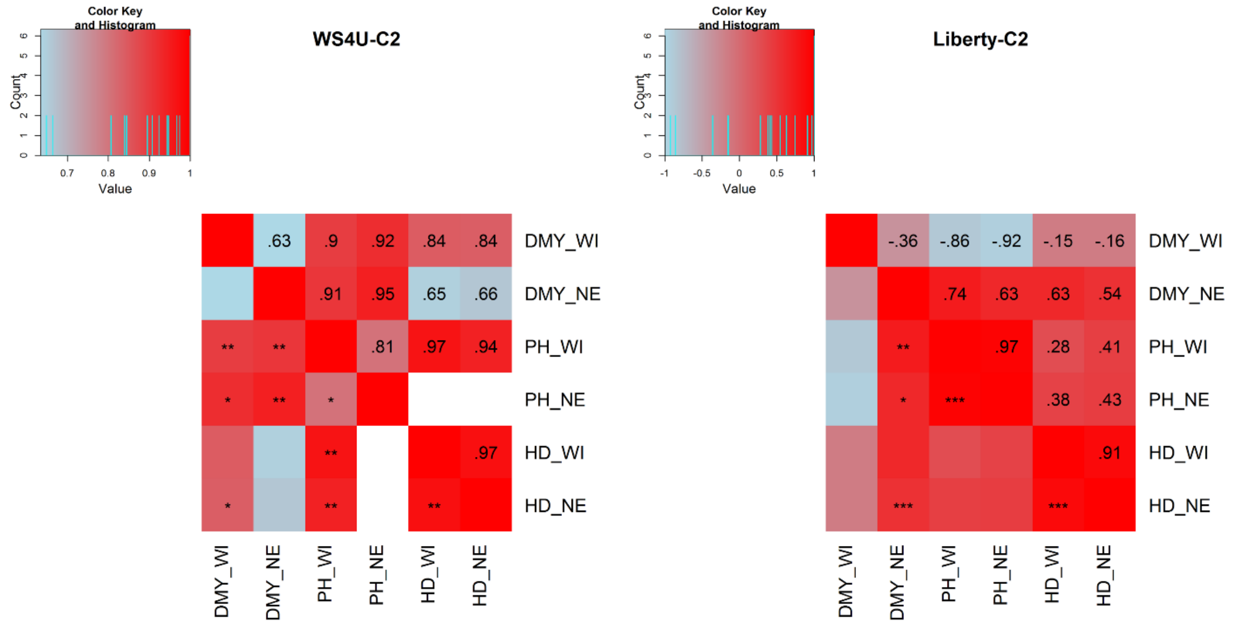

Figure S3 – *Heatmaps of genomic correlations between trait BLUPs for traits measured (a) in WS4U-C2; (b) in Liberty-C2. White cells show unavailable estimates (due to non-convergence of the fitting algorithm). Significance of inferred genotypic correlations was assessed by likelihood-ratio tests; *: $p < 0.05$, **: $p < 0.01$, ***: $p < 0.001$. Genomic correlations were estimated in a multivariate GBLUP model, with $\mathbf{X}_{Base}$ as input, using ASREML-R (Butler et al. 2007).*

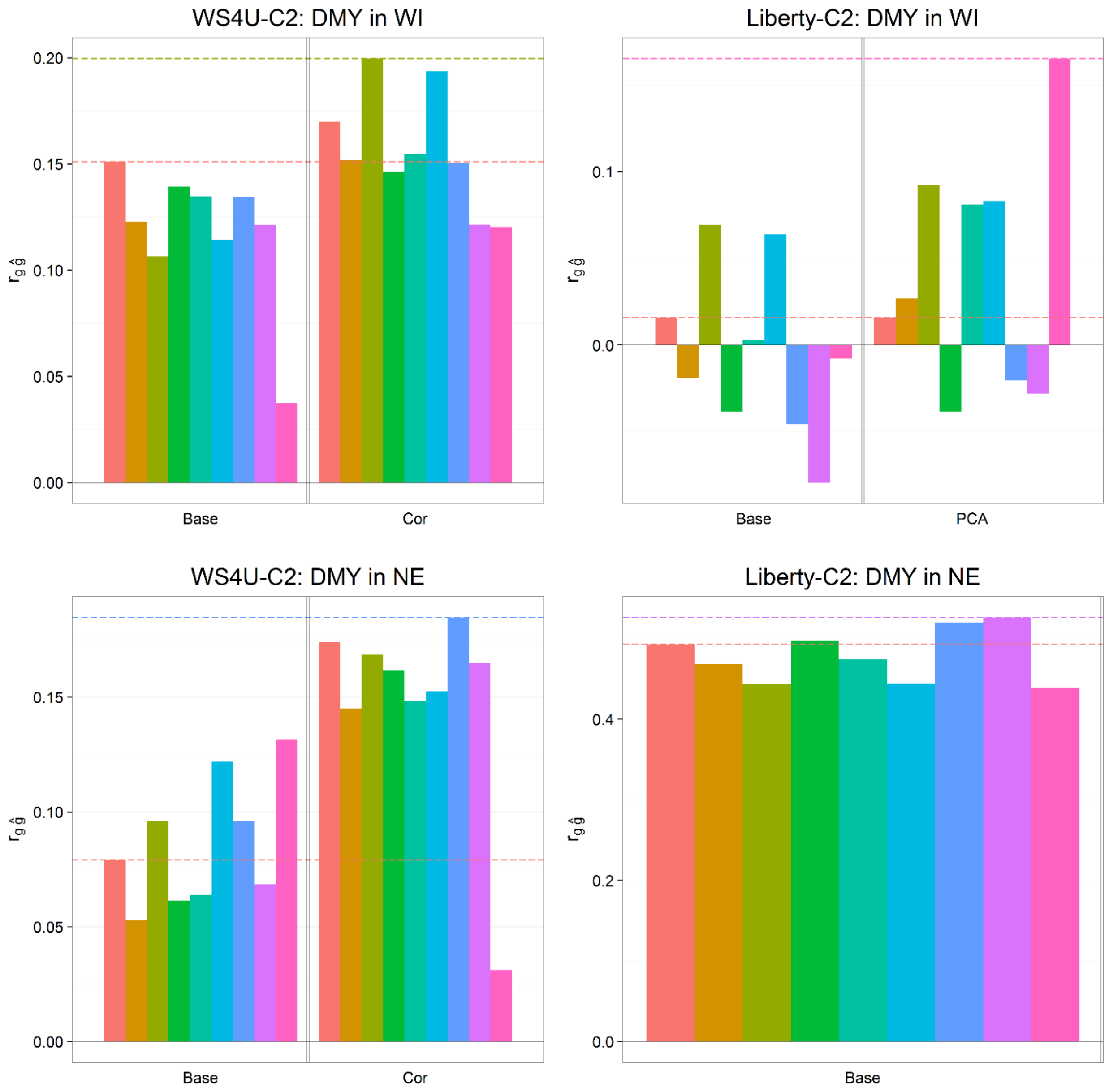
WS4U-C2: DMY in WI
Liberty-C2: DMY in WI
WS4U-C2: DMY in NE
Liberty-C2: DMY in NE
$r_{g\hat{g}}$
0.20
0.15
0.10
0.05
0.00
0.1
0.0
0.4
0.2
Base
Cor
PCA

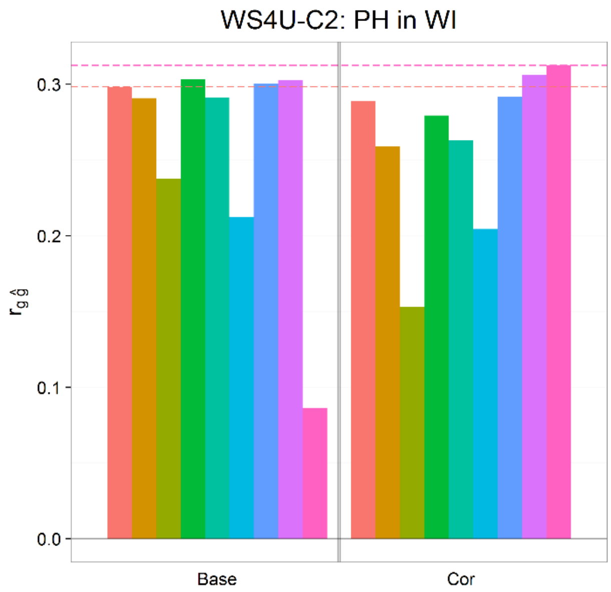
WS4U-C2: PH in WI
0.3
0.2
0.1
0.0
Base
Cor

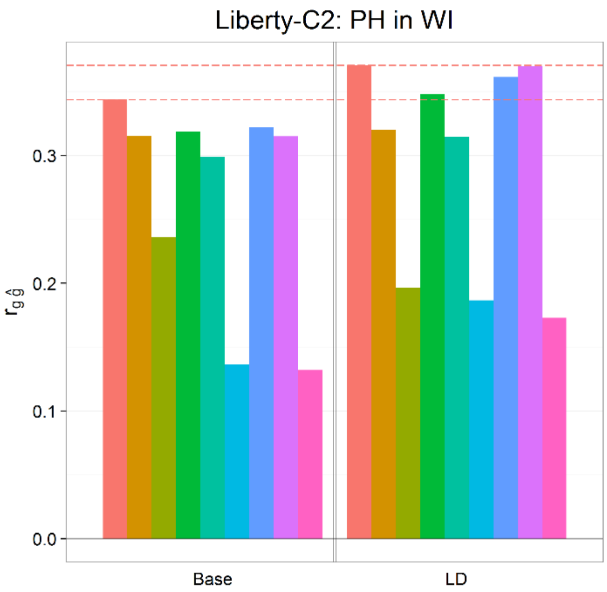
Liberty-C2: PH in WI
0.3
0.2
0.1
0.0
Base
LD

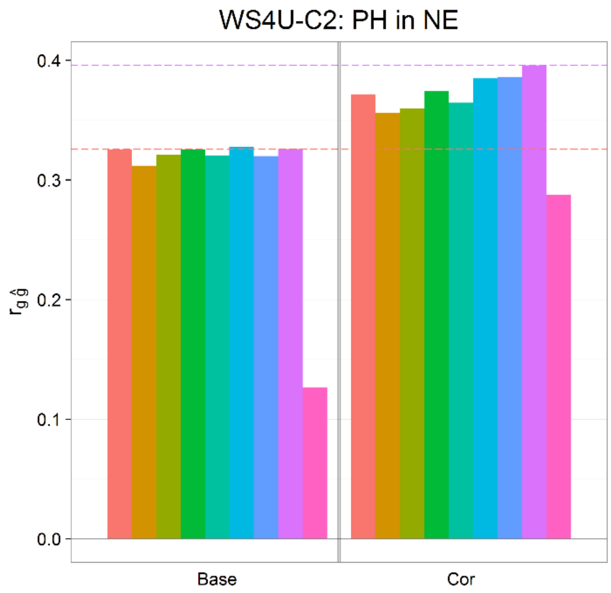
WS4U-C2: PH in NE
0.4
0.3
0.2
0.1
0.0
Base
Cor

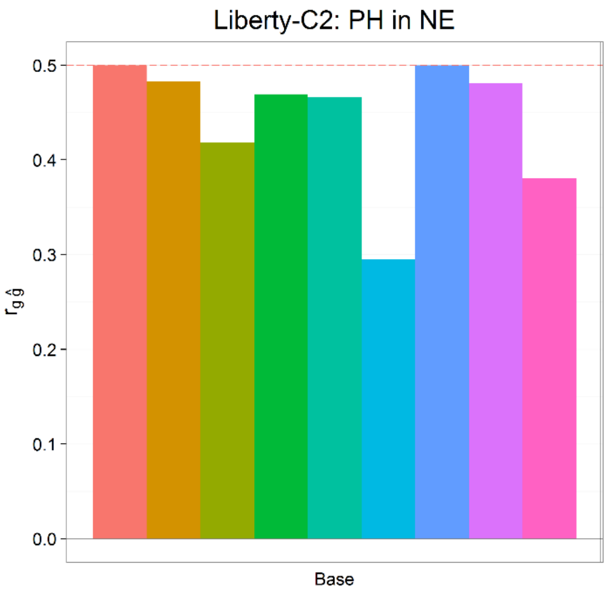
Liberty-C2: PH in NE
0.5
0.4
0.3
0.2
0.1
0.0
Base

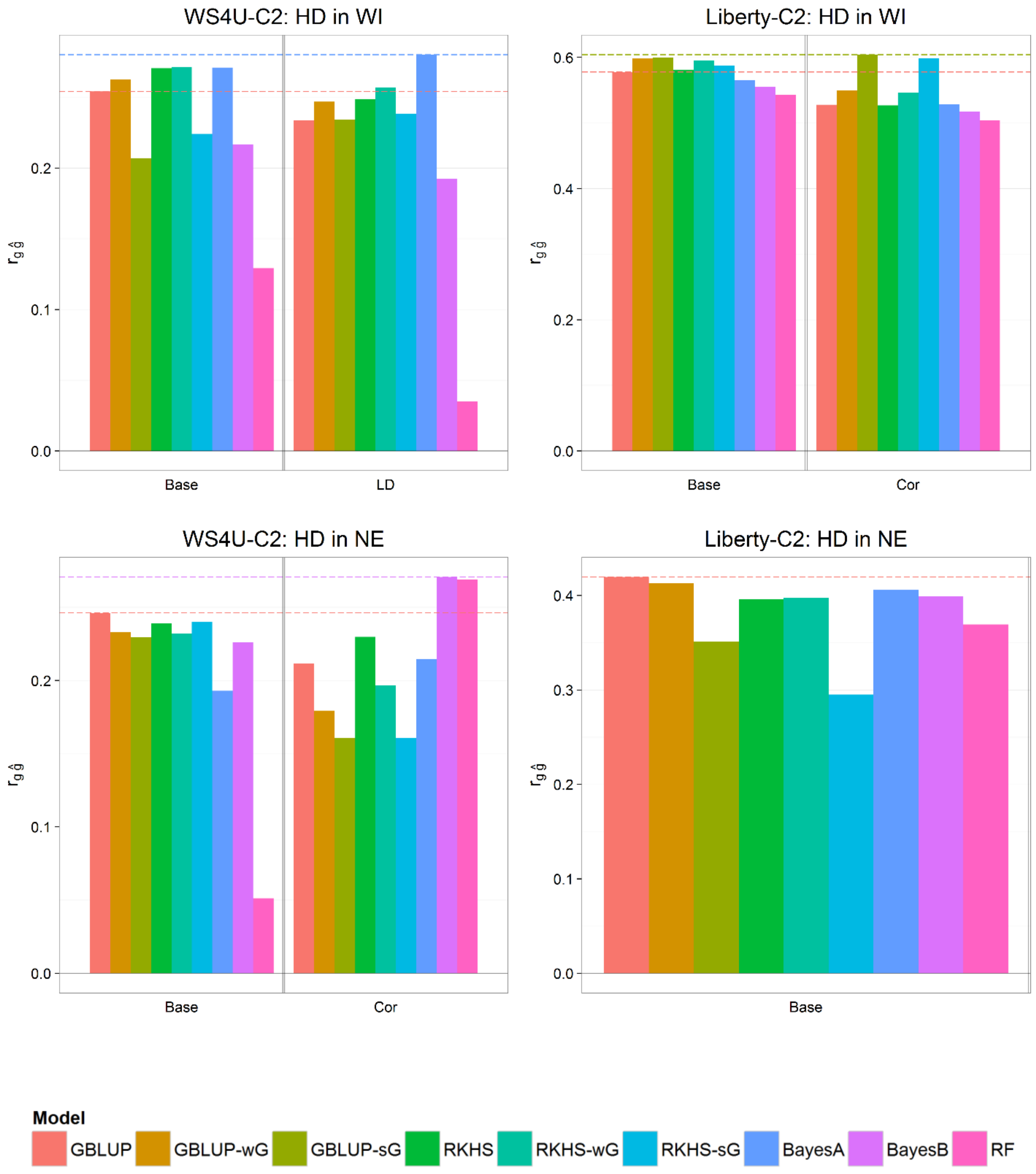

Figure S4 – *Bar plots of mean prediction accuracies from non-replicated five-fold cross-validation for all outcomes. The values showed correspond to those in Tables 3 and S3. For a given outcome, only the values for Base and the selected marker-data transformation are shown.*

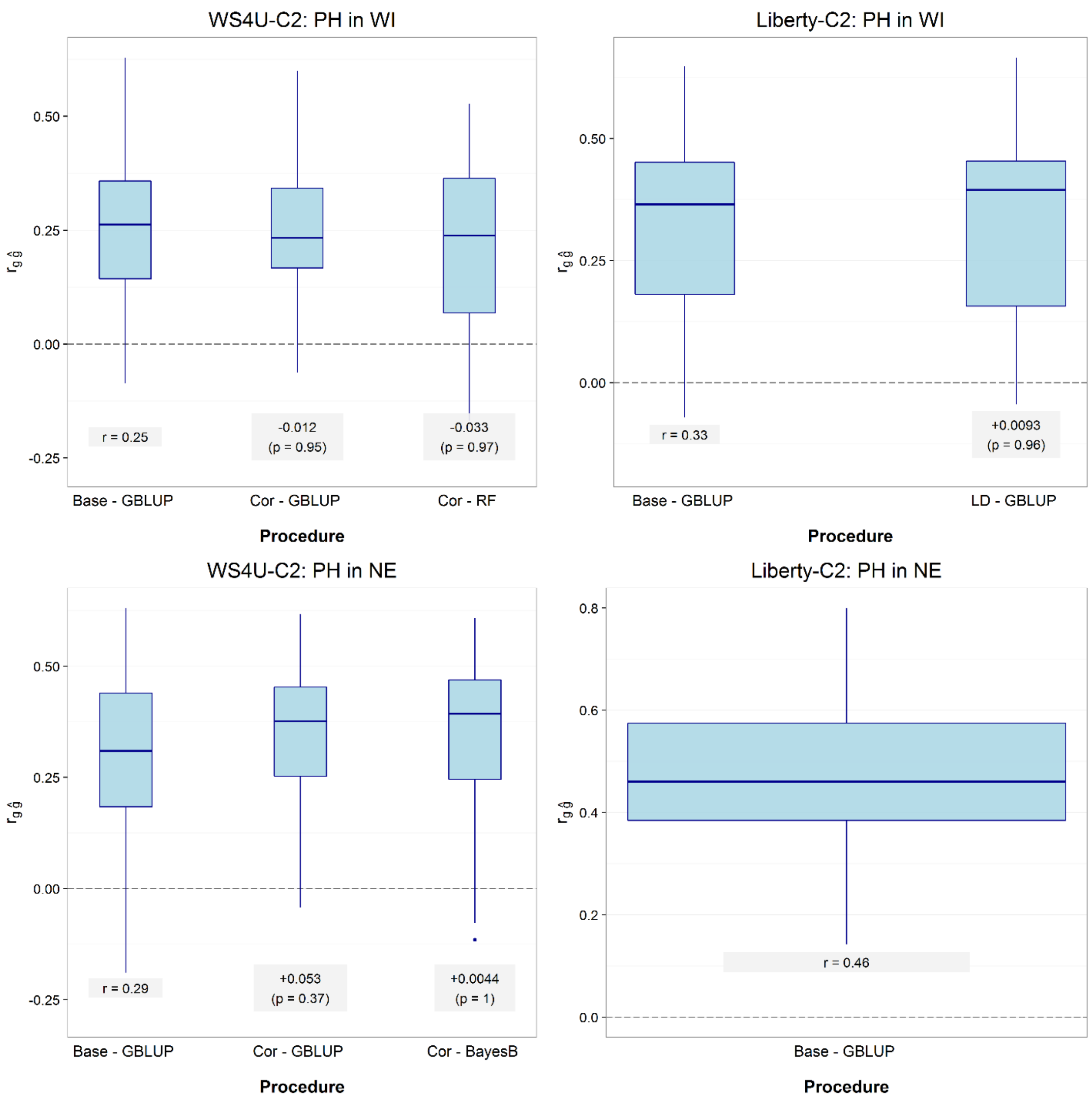
WS4U-C2: PH in WI
Liberty-C2: PH in WI
WS4U-C2: PH in NE
Liberty-C2: PH in NE
r = 0.25
-0.012
(p = 0.95)
-0.033
(p = 0.97)
r = 0.33
+0.0093
(p = 0.96)
r = 0.29
+0.053
(p = 0.37)
+0.0044
(p = 1)
r = 0.46
Base - GBLUP
Cor - GBLUP
Cor - RF
LD - GBLUP
Cor - BayesB
Procedure
0.50
0.25
0.00
-0.25
0.8
0.6
0.4
0.2
0.0

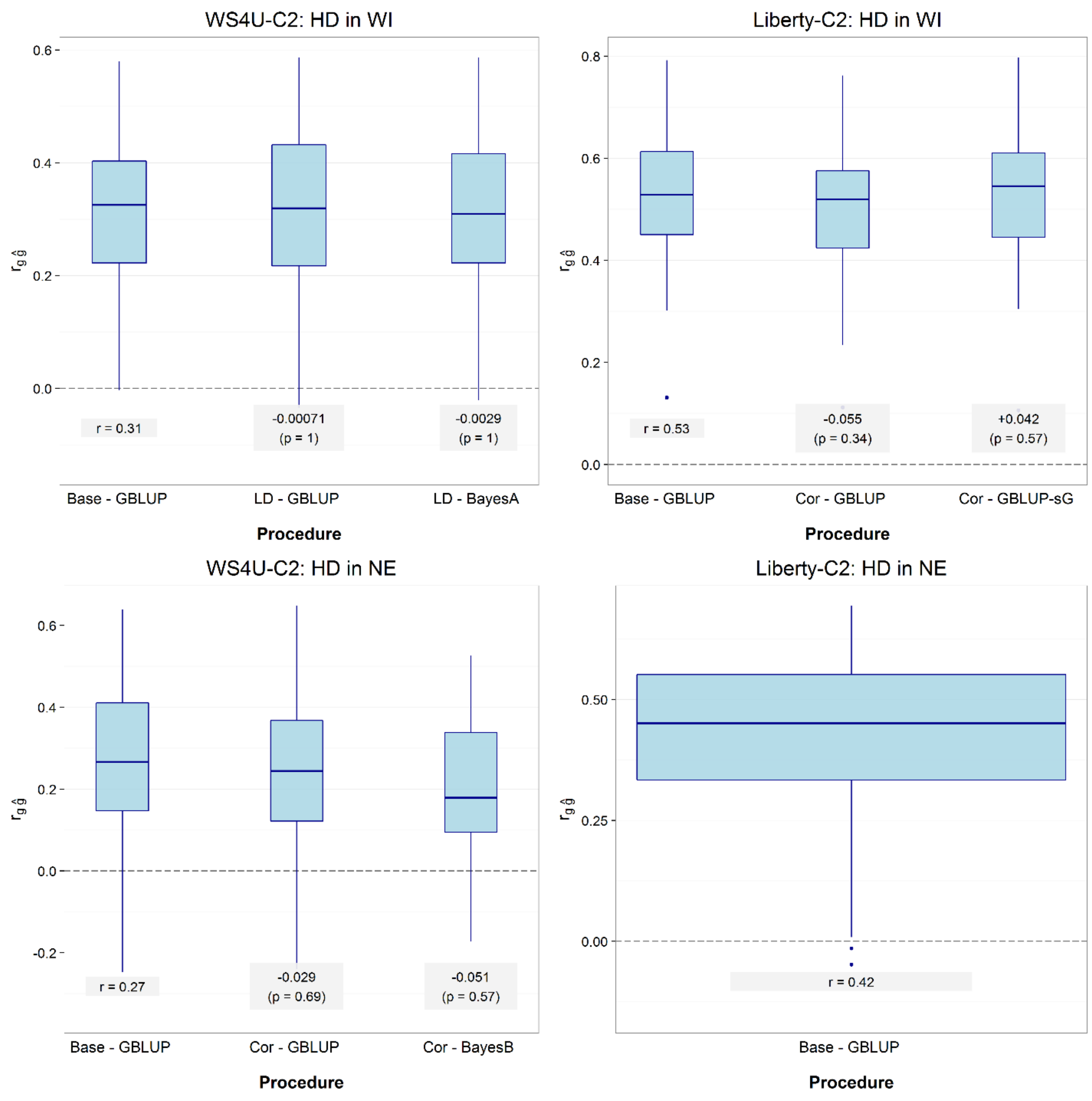

Figure S5 – *Validation of selected prediction procedures for PH and HD in WI and NE. Prediction accuracies ($r_{g\hat{g}}$) were estimated with a within-population/within-environment learning scheme in five-fold cross-validation, replicated ten times. In each boxplot, up to two comparisons are made: (i) the candidate-transformation procedure (selected marker-data transformation according to non-replicated five-fold cross-validation in a GBLUP model; Table S3) is compared to the standard procedure (Base – GBLUP) – if relevant; and (ii) the candidate procedure (selected prediction procedure according to non-replicated five-fold cross-validation; Table S3) is compared to the candidate-transformation procedure. The significance of differences in prediction accuracies was assessed by two-sided paired Dunnett tests, which accounted for multiple testing of data*

transformations, in (i), and of prediction models, in (ii). The t-statistics in Dunnett tests were adjusted to account for correlation among training sets in cross-validation, as described in Bouckaert and Frank (2004).

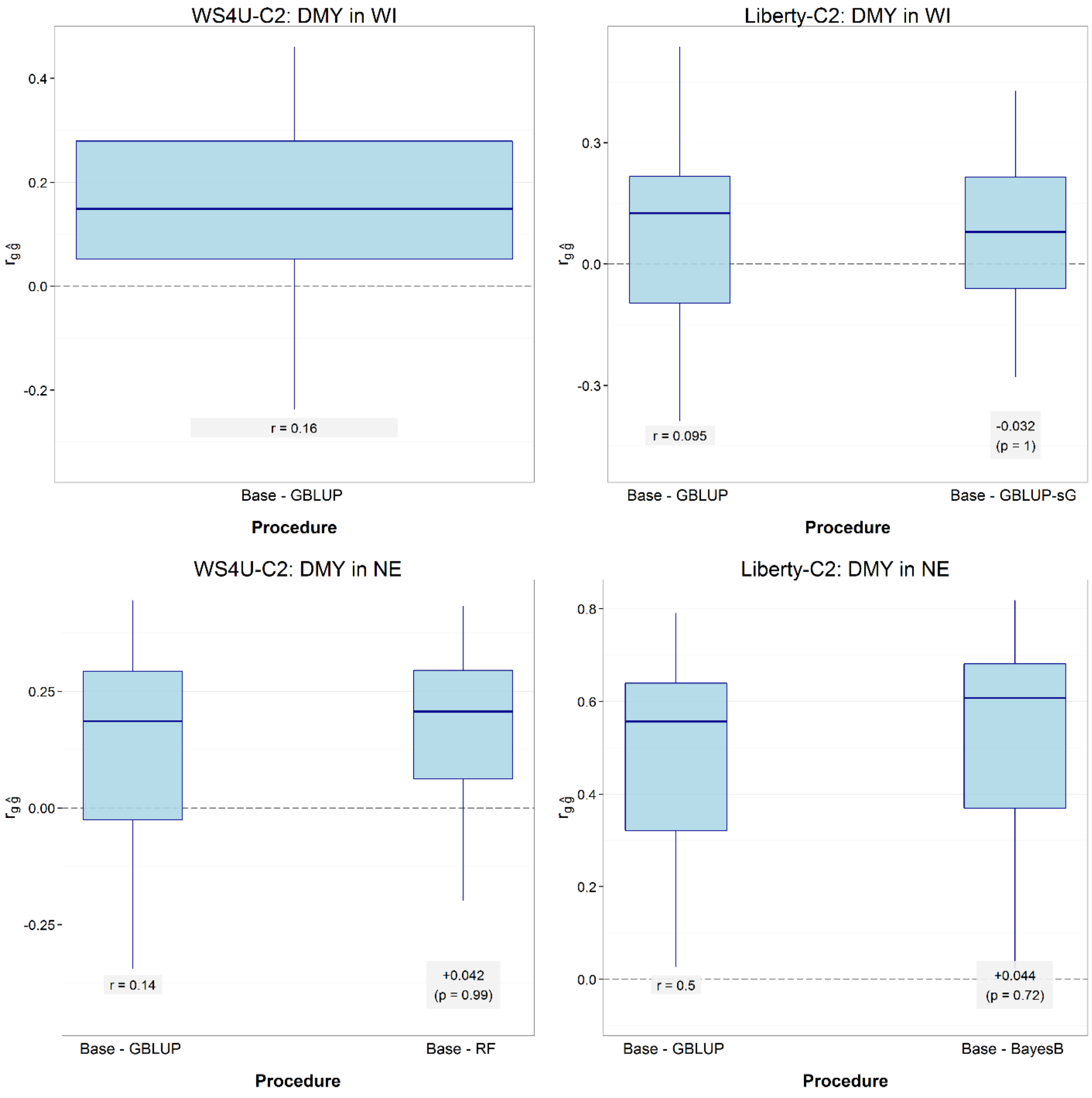
WS4U-C2: DMY in WI
0.4
0.2
0.0
-0.2
$r_{g\hat{g}}$
r = 0.16
Base - GBLUP
Procedure
Liberty-C2: DMY in WI
0.3
0.0
-0.3
$r_{g\hat{g}}$
r = 0.095
-0.032
(p = 1)
Base - GBLUP
Base - GBLUP-sG
Procedure
WS4U-C2: DMY in NE
0.25
0.00
-0.25
$r_{g\hat{g}}$
r = 0.14
+0.042
(p = 0.99)
Base - GBLUP
Base - RF
Procedure
Liberty-C2: DMY in NE
0.8
0.6
0.4
0.2
0.0
$r_{g\hat{g}}$
r = 0.5
+0.044
(p = 0.72)
Base - GBLUP
Base - BayesB
Procedure

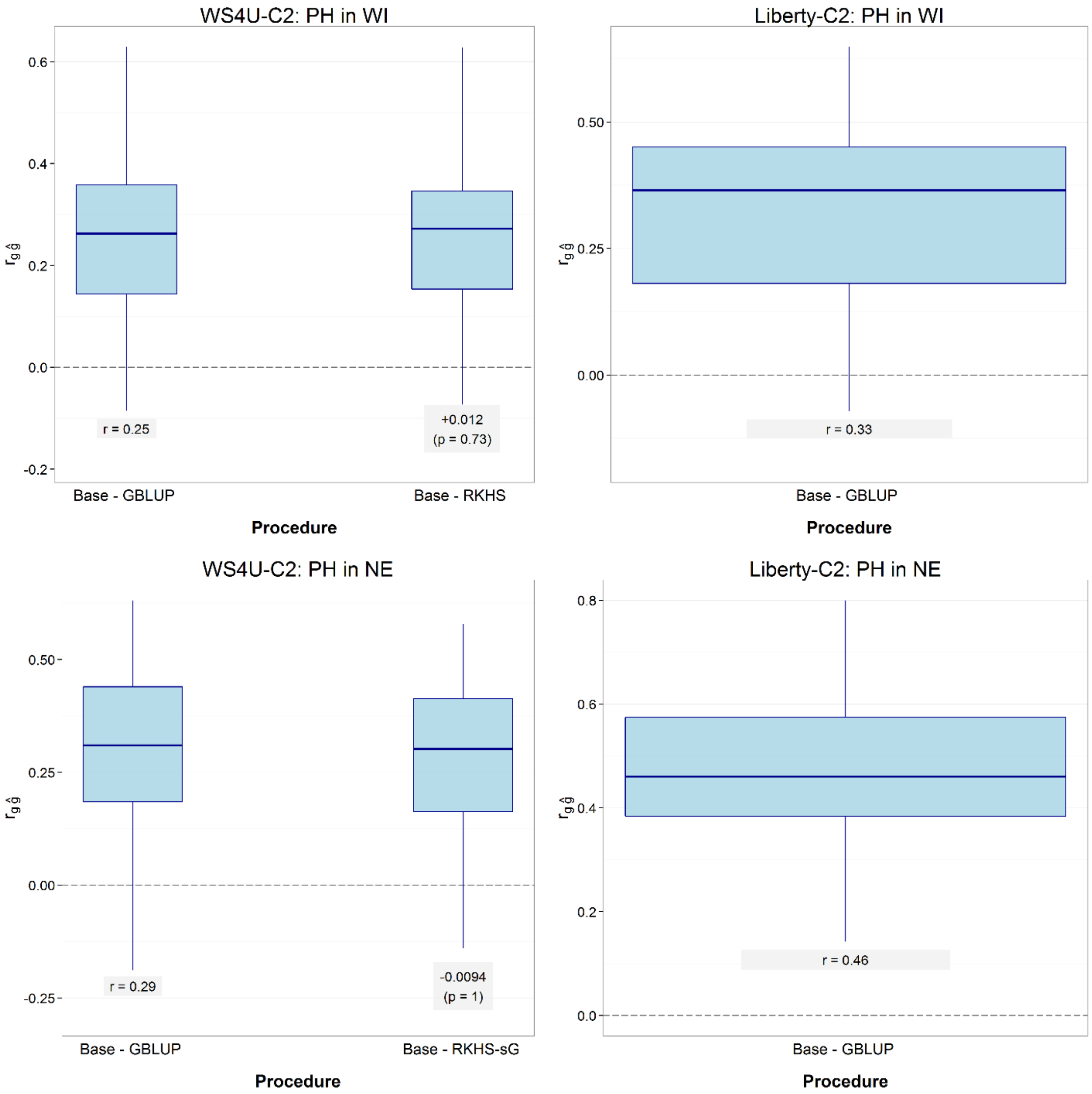

WS4U-C2: PH in WI
0.6
0.4
0.2
0.0
-0.2
$r_{g\hat{g}}$
r = 0.25
+0.012
(p = 0.73)
Base - GBLUP
Base - RKHS
Procedure
Liberty-C2: PH in WI
0.50
0.25
0.00
r = 0.33
Base - GBLUP
Procedure
WS4U-C2: PH in NE
0.50
0.25
0.00
-0.25
r = 0.29
-0.0094
(p = 1)
Base - GBLUP
Base - RKHS-sG
Procedure
Liberty-C2: PH in NE
0.8
0.6
0.4
0.2
0.0
r = 0.46
Base - GBLUP
Procedure

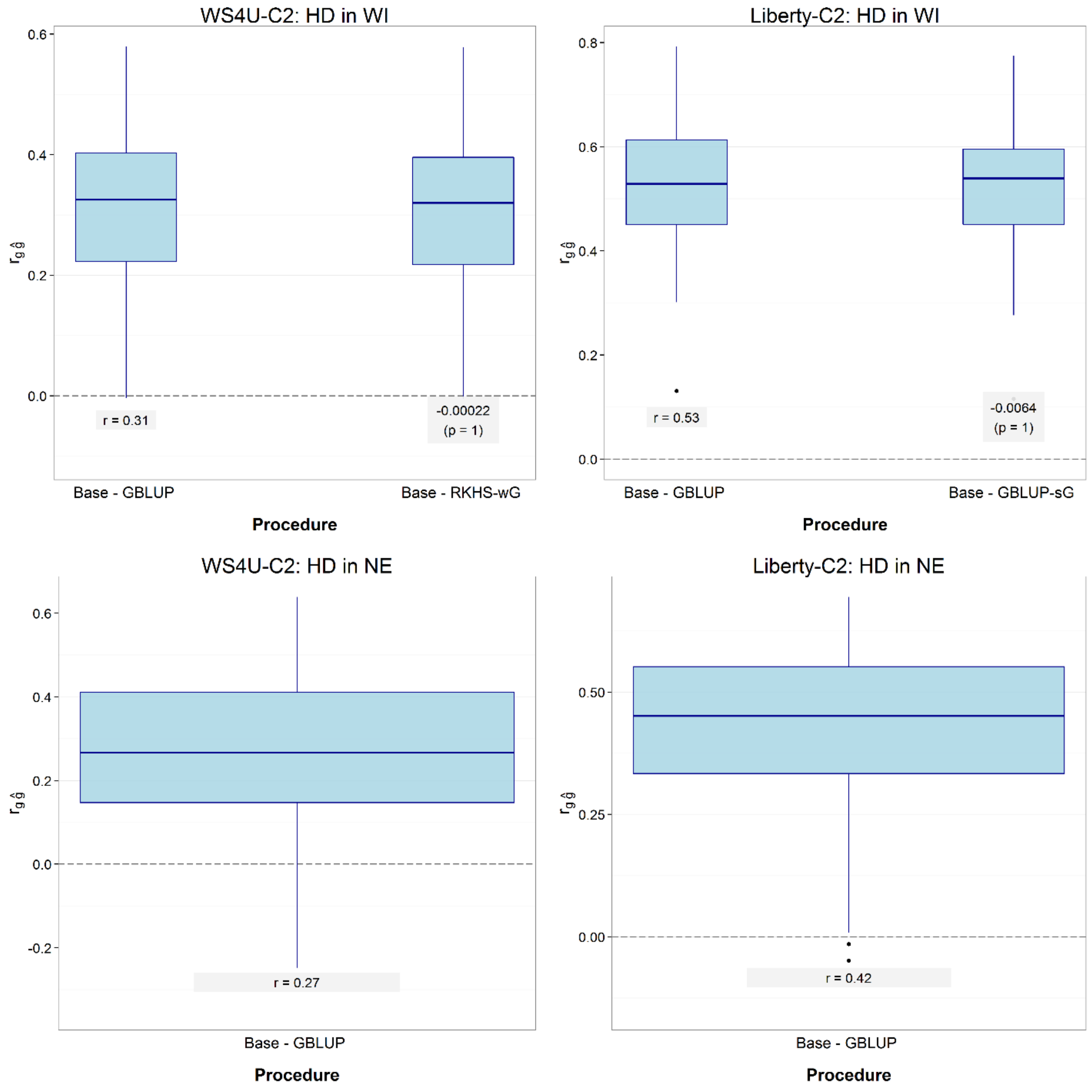

Figure S6 – *Validation of selected prediction procedures for all outcomes, ignoring all alternate marker-data transformations (i.e., other than Base). Prediction accuracies ($r_{g\hat{g}}$) were estimated with a within-population/within-environment learning scheme in five-fold cross-validation, replicated ten times. In each boxplot, the selected procedure (best prediction procedure according to non-replicated five-fold cross-validation; Tables 3 and S3) is compared to the standard procedure (Base – GBLUP). The significance of differences in prediction accuracies was assessed by two-sided paired Dunnett tests, which accounted for multiple testing of data transformations of prediction models. The t-statistics in Dunnett tests were adjusted to account for correlation among training sets in cross-validation, as described in Bouckaert and Frank (2004).*

File S1: Raw phenotypic data

Excel (.xls) file consisting of two sheets: one for traits measured at the plant level (Heading date – in day-of-year – and Plant height – in cm) and the other for traits measured at the plot level (Fresh biomass yield, Sample fresh matter weight and Sample dry matter weight – in grams).

Population: WS4U-C2 or Liberty-C2
Genotype: genotype index for female parent within Population
Row: row number (as coordinate)
Col: rolumn number (as coordinate)
Rep: replicate
Plant (for plant-level traits): plant index within plot
Number_of_plants (for plot-level traits): number of (surviving) plants within plot
Location: ARL (Arlington, WI) or MEAD (Mead, NE)
Year: 2012, 2013 or 2014

Plots are combinations of Location, Genotype and Rep.

In this study, dry matter yield was calculated as:
(Fresh biomass yield) * (Sample dry matter weight) / (Sample fresh matter weight).

File S2: Half-sib (HS) family BLUPs

Excel (.xls) file containing best linear unbiased predictions (predicted random effects) of HS family effects as obtained from the models described in the article, fitted for each population and outcome separately.

Population: WS4U-C2 or Liberty-C2
Genotype: genotype index for HS-family parent within Population
PH_WI, HD_WI, DMY_WI: Plant height, Heading date and Dry matter yield, respectively, in Arlington, WI.
PH_NE, HD_NE, DMY_NE: Plant height, Heading date and Dry matter yield, respectively, in Mead, NE.

File S3: Untransformed genotype calls at marker loci

Compression (.rar file) of a .csv file with expected allelic dosages (expected number of alternate alleles, as per posterior probabilities from genotype calling), rounded to the fifth decimal digit, at selected markers for each genotype (HS-family parent). Rows correspond to the q*=141,030 marker loci selected across both populations (see main text) and columns correspond to all 247 genotypes across both populations. Note that a .xlsx version of this file, with non-rounded values, is available from http://dfrc.wisc.edu/sniper/.

Row name: Chromosome index + SNP index (e.g. 'Chr05bSNP1187770' refers to SNP 1187770 in chromosome 5, subgenome B)
Column name: Population name + genotype index (e.g. 'Liberty-C2_31804' refers to genotype 31804 in population Liberty-C2)
