# Supplementary material for: Accuracy of Genomic Prediction in Switchgrass (Panicum virgatum L.) Improved by Accounting for Linkage Disequilibrium
Source: G3 (Bethesda). 2016 Feb 10;6(4):1049–62. doi: 10.1534/g3.115.024950 (PMC4825640; doi:10.1534/g3.115.024950)
Supplement: Supplemental Material [file supp_g3.115.024950_FigureS1.pdf]

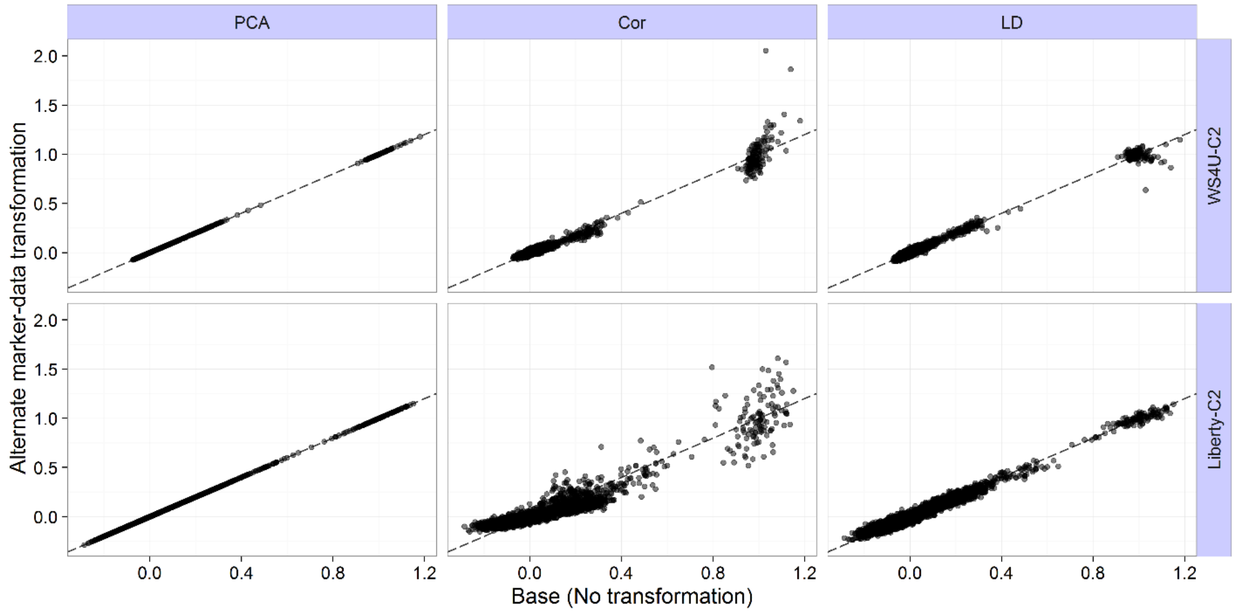

Figure S1 – *Genetic relationship coefficients in WS4U-C2 (upper panel) and Liberty-C2 (lower panel), based on marker features from alternate data transformations (PCA, Cor or LD; see section Material and methods), compared to Base (only centering of expected allelic dosages).*
