# Supplementary material for: Accuracy of Genomic Prediction in Switchgrass (Panicum virgatum L.) Improved by Accounting for Linkage Disequilibrium
Source: G3 (Bethesda). 2016 Feb 10;6(4):1049–62. doi: 10.1534/g3.115.024950 (PMC4825640; doi:10.1534/g3.115.024950)
Supplement: Supplemental Material [file supp_g3.115.024950_FigureS2.pdf]

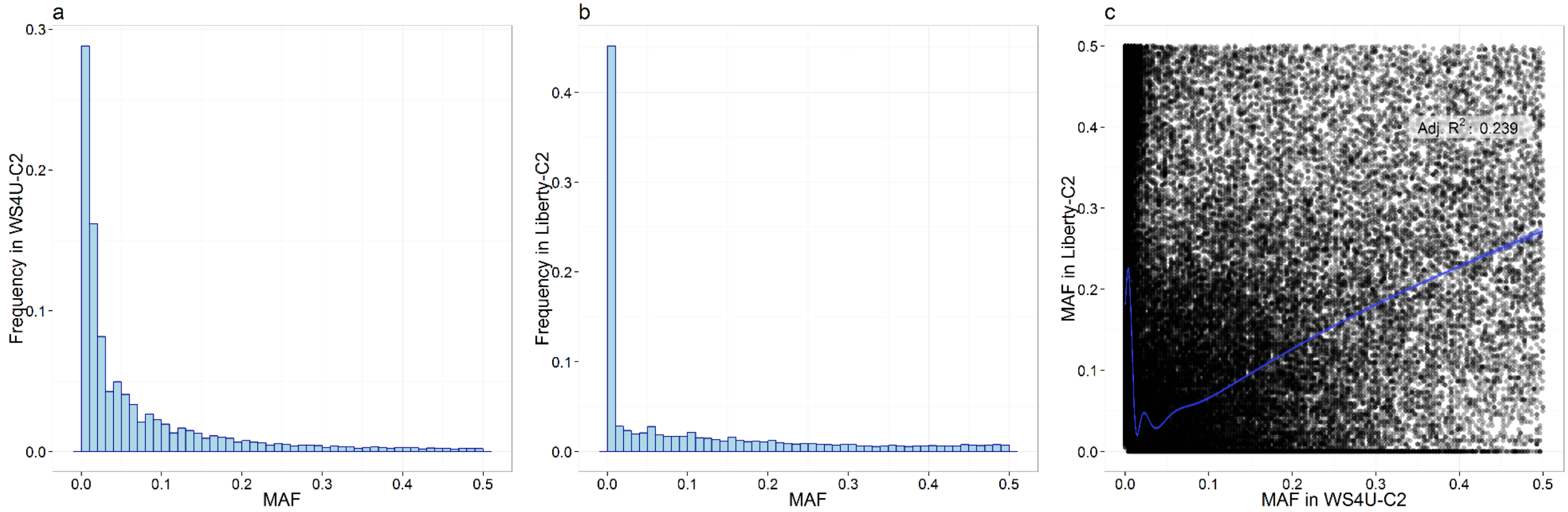

Figure S2. Distribution of minor allele frequency (MAF) in (a) WS4U-C2 and (b) Liberty-C2; (c) Concordance of MAF from WS4U-C2 to Liberty-C2; the blue curve corresponds to the mean value (and its 95%-confidence interval) from a cubic-regression spline model assuming a Normal distribution for MAF in Liberty-C2. Cubic-regression spline models were fitted using the R package mgcv (Wood 2006).
