# Supplementary material for: Accuracy of Genomic Prediction in Switchgrass (Panicum virgatum L.) Improved by Accounting for Linkage Disequilibrium
Source: G3 (Bethesda). 2016 Feb 10;6(4):1049–62. doi: 10.1534/g3.115.024950 (PMC4825640; doi:10.1534/g3.115.024950)
Supplement: Supplemental Material [file supp_g3.115.024950_FigureS3.pdf]

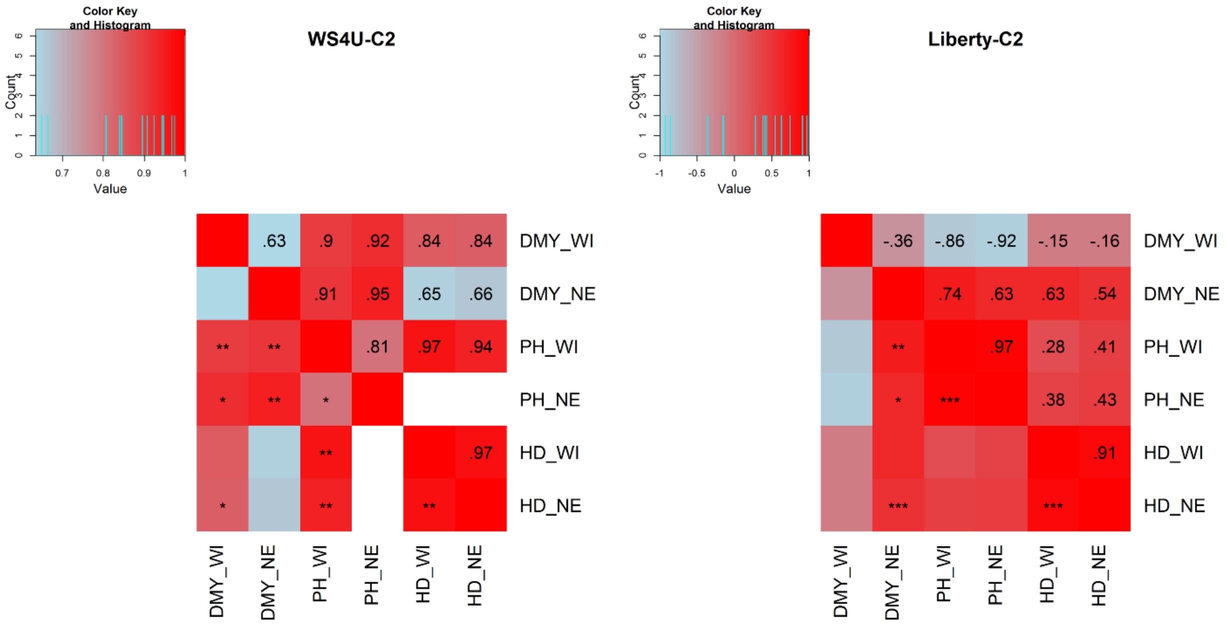

Figure S3. Heatmaps of genomic correlations between trait BLUPs for traits measured (a) in WS4U-C2; (b) in Liberty-C2.

White cells show unavailable estimates (due to non-convergence of the fitting algorithm). Significance of inferred genotypic correlations was assessed by likelihood-ratio tests; *: $p < 0.05$, **: $p < 0.01$, ***: $p < 0.001$. Genomic correlations were estimated in a multivariate GBLUP model, with $\mathbf{X}_{\text{Base}}$ as input, using ASREML-R (Butler et al. 2007).
