# Supplementary material for: Accuracy of Genomic Prediction in Switchgrass (Panicum virgatum L.) Improved by Accounting for Linkage Disequilibrium
Source: G3 (Bethesda). 2016 Feb 10;6(4):1049–62. doi: 10.1534/g3.115.024950 (PMC4825640; doi:10.1534/g3.115.024950)
Supplement: Supplemental Material [file supp_g3.115.024950_FigureS4.pdf]

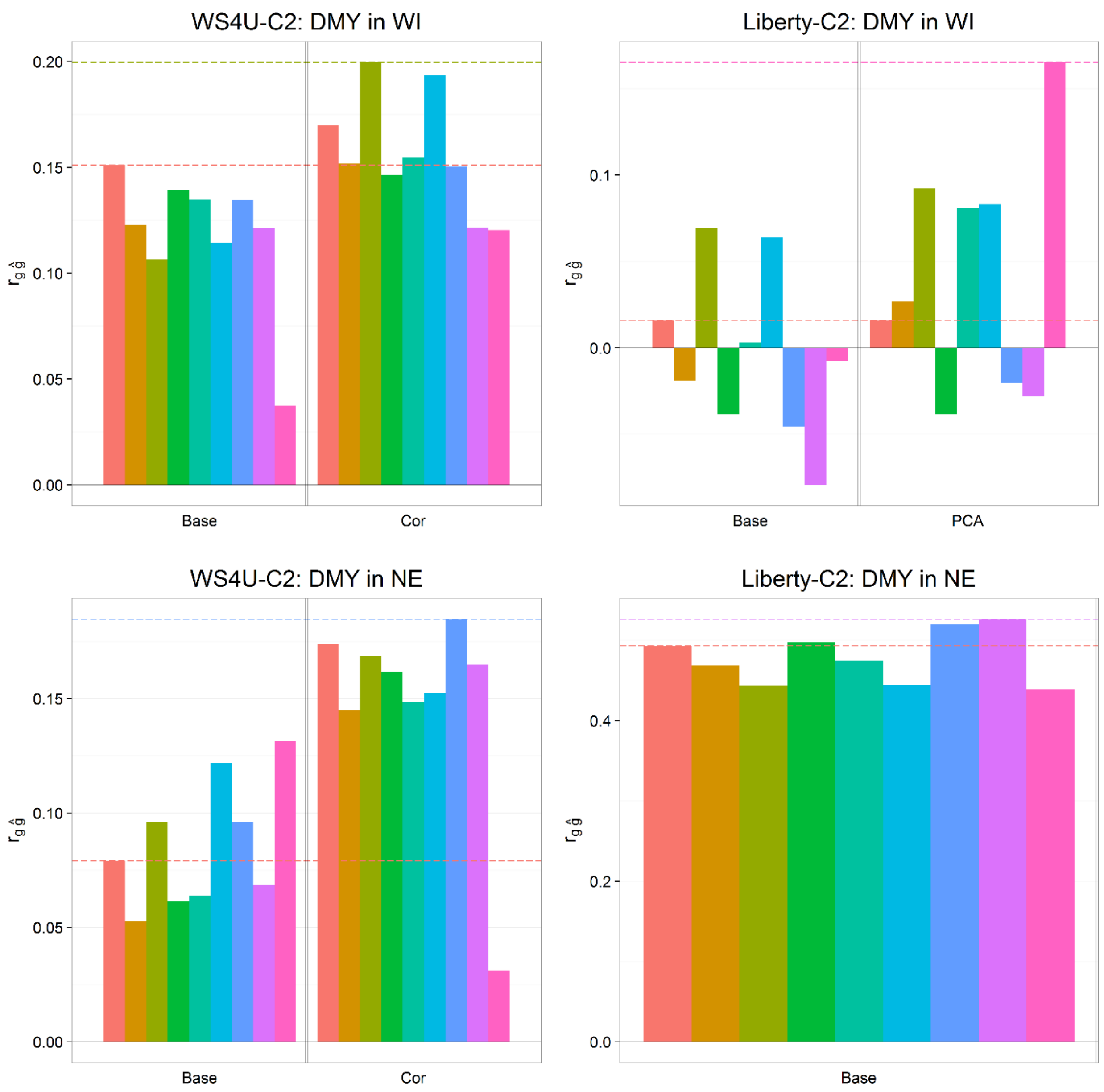

WS4U-C2: DMY in WI
Liberty-C2: DMY in WI
WS4U-C2: DMY in NE
Liberty-C2: DMY in NE
$r_{g\hat{g}}$
0.00
0.05
0.10
0.15
0.20
0.0
0.1
0.2
0.4
Base
Cor
PCA

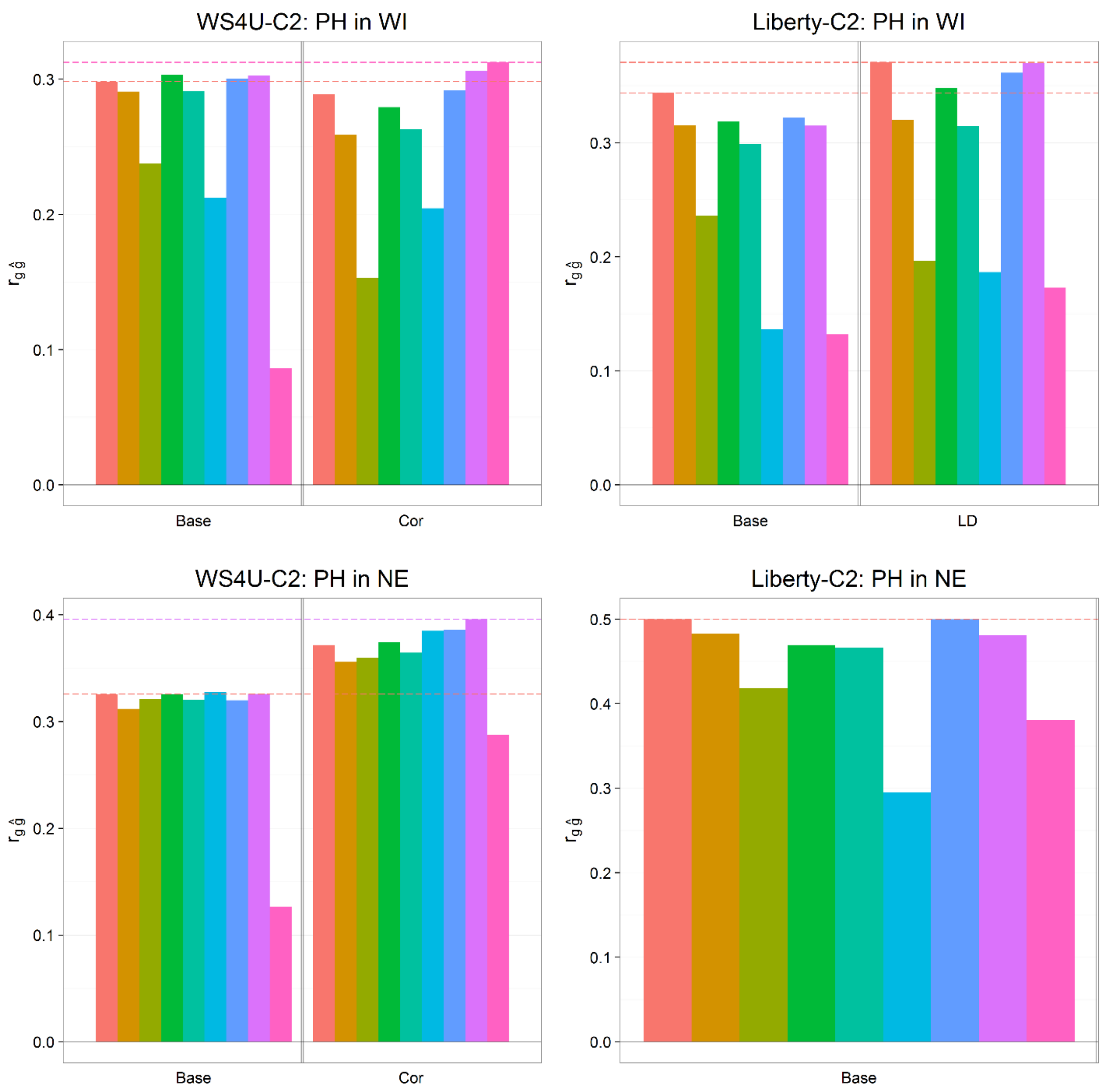

WS4U-C2: PH in WI
Liberty-C2: PH in WI
WS4U-C2: PH in NE
Liberty-C2: PH in NE
$r_{g\hat{g}}$
0.0
0.1
0.2
0.3
0.4
0.5
Base
Cor
LD

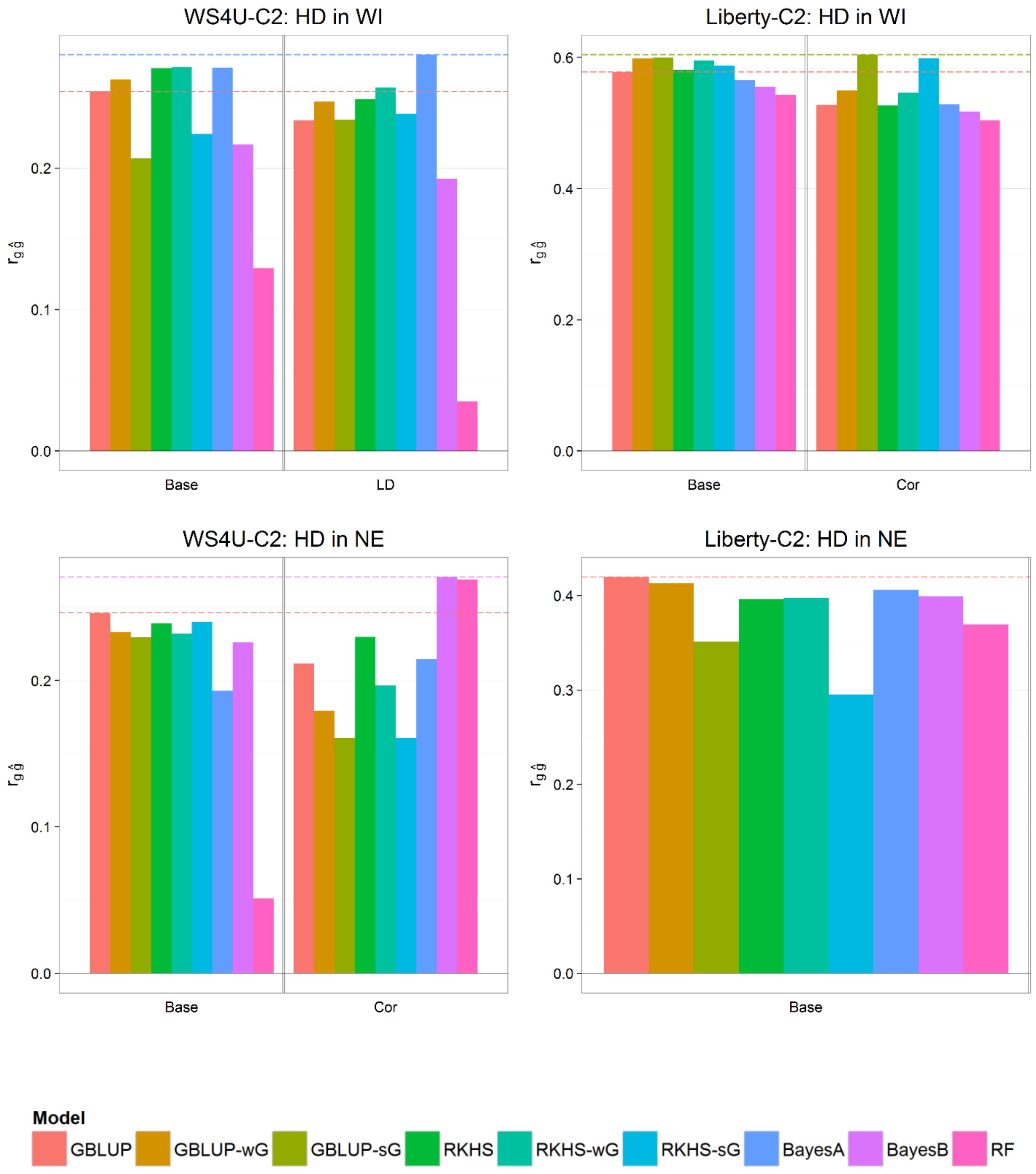

Figure S4 – *Bar plots of mean prediction accuracies from non-replicated five-fold cross-validation for all outcomes. The values showed correspond to those in Tables 3 and S3. For a given outcome, only the values for Base and the selected marker-data transformation are shown.*
