# Supplementary material for: Accuracy of Genomic Prediction in Switchgrass (Panicum virgatum L.) Improved by Accounting for Linkage Disequilibrium
Source: G3 (Bethesda). 2016 Feb 10;6(4):1049–62. doi: 10.1534/g3.115.024950 (PMC4825640; doi:10.1534/g3.115.024950)
Supplement: Supplemental Material [file supp_g3.115.024950_FigureS5.pdf]

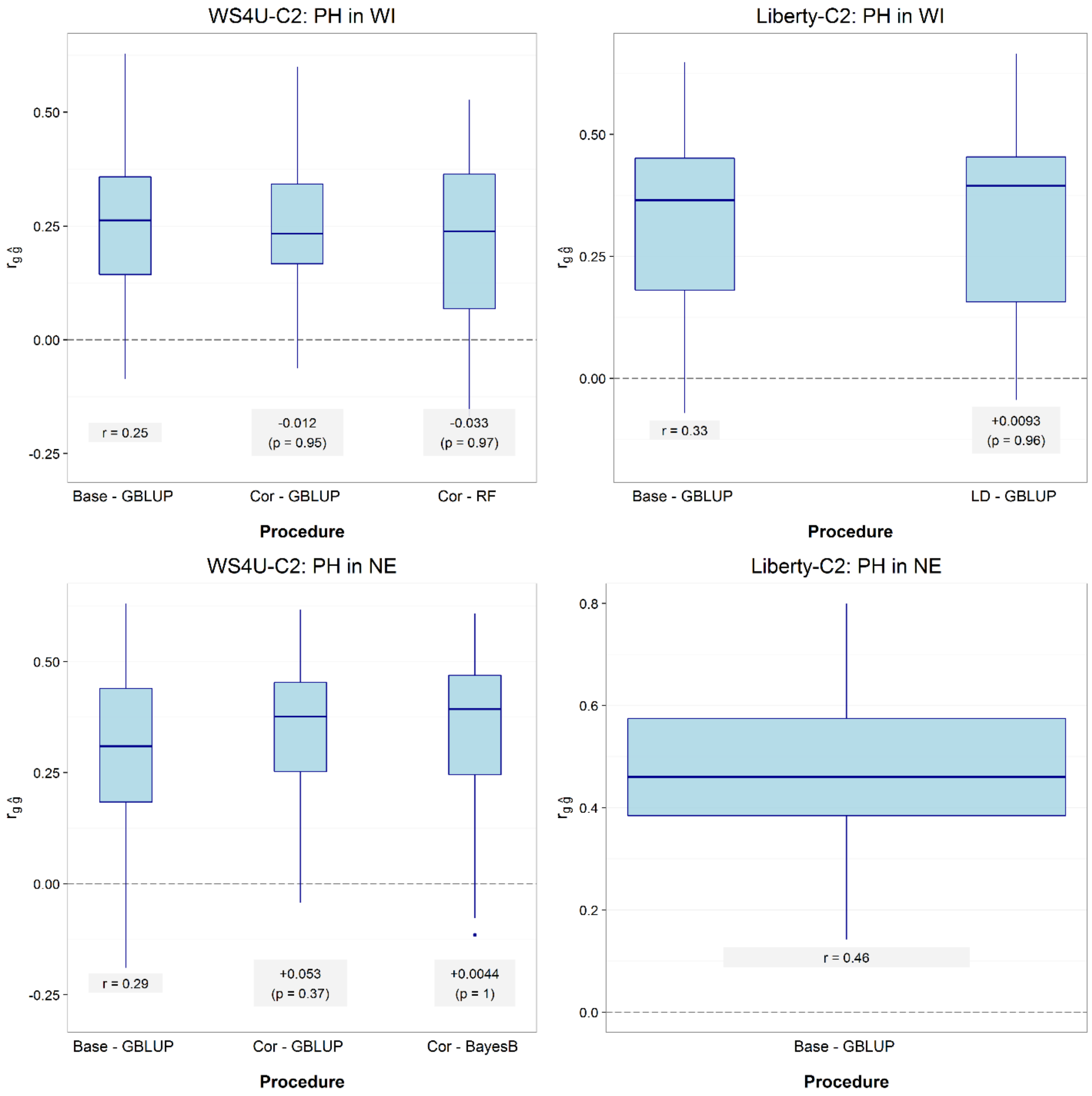
WS4U-C2: PH in WI
Liberty-C2: PH in WI
WS4U-C2: PH in NE
Liberty-C2: PH in NE
0.50
0.25
0.00
-0.25
0.8
0.6
0.4
0.2
0.0
r = 0.25
-0.012
(p = 0.95)
-0.033
(p = 0.97)
r = 0.33
+0.0093
(p = 0.96)
r = 0.29
+0.053
(p = 0.37)
+0.0044
(p = 1)
r = 0.46
Base - GBLUP
Cor - GBLUP
Cor - RF
LD - GBLUP
Cor - BayesB
Procedure

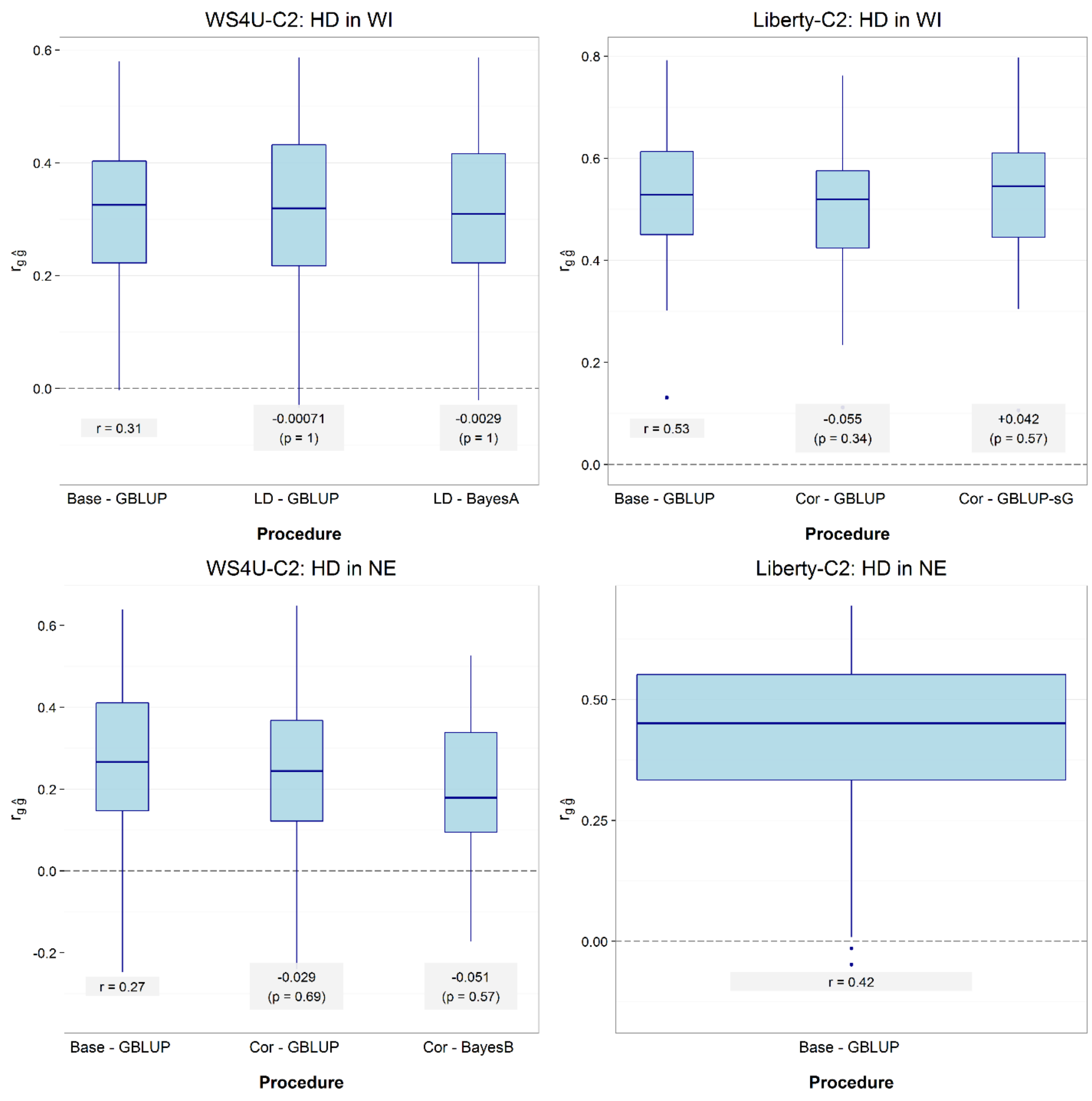

Figure S5 – *Validation of selected prediction procedures for PH and HD in WI and NE. Prediction accuracies ($r_{g\hat{g}}$) were estimated with a within-population/within-environment learning scheme in five-fold cross-validation, replicated ten times. In each boxplot, up to two comparisons are made: (i) the candidate-transformation procedure (selected marker-data transformation according to non-replicated five-fold cross-validation in a GBLUP model; Table S3) is compared to the standard procedure (Base – GBLUP) – if relevant; and (ii) the candidate procedure (selected prediction procedure according to non-replicated five-fold cross-validation; Table S3) is compared to the candidate-transformation procedure. The significance of differences in prediction accuracies was assessed by two-sided paired Dunnett tests, which accounted for multiple testing of data*

transformations, in (i), and of prediction models, in (ii). The t-statistics in Dunnett tests were adjusted to account for correlation among training sets in cross-validation, as described in Bouckaert and Frank (2004).
