# Supplementary material for: Accuracy of Genomic Prediction in Switchgrass (Panicum virgatum L.) Improved by Accounting for Linkage Disequilibrium
Source: G3 (Bethesda). 2016 Feb 10;6(4):1049–62. doi: 10.1534/g3.115.024950 (PMC4825640; doi:10.1534/g3.115.024950)
Supplement: Supplemental Material [file supp_g3.115.024950_FigureS6.pdf]

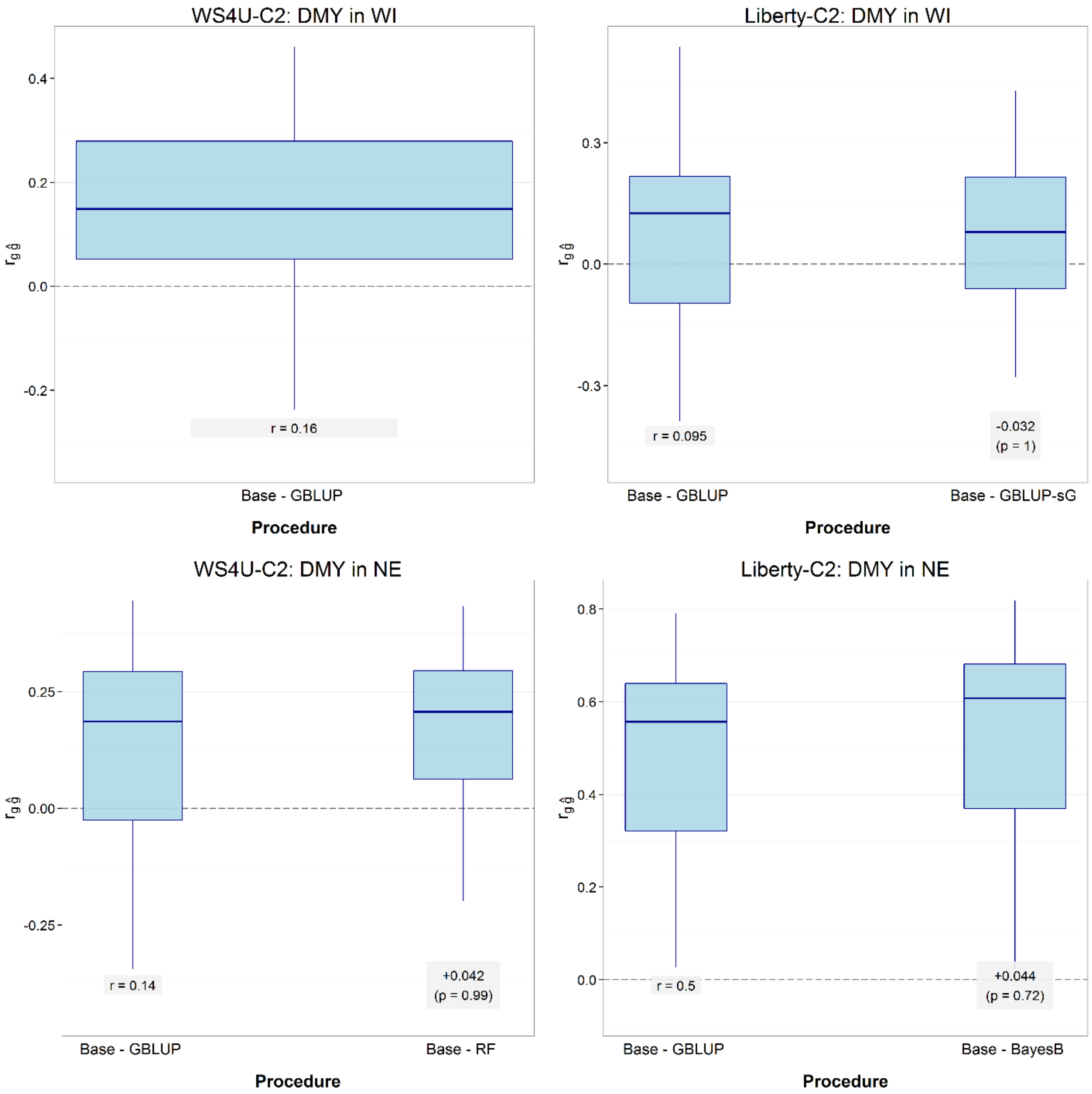

WS4U-C2: DMY in WI
0.4
0.2
0.0
-0.2
$r_{g\hat{g}}$
r = 0.16
Base - GBLUP
Procedure
Liberty-C2: DMY in WI
0.3
0.0
-0.3
r = 0.095
-0.032
(p = 1)
Base - GBLUP
Base - GBLUP-sG
Procedure
WS4U-C2: DMY in NE
0.25
0.00
-0.25
r = 0.14
+0.042
(p = 0.99)
Base - GBLUP
Base - RF
Procedure
Liberty-C2: DMY in NE
0.8
0.6
0.4
0.2
0.0
r = 0.5
+0.044
(p = 0.72)
Base - GBLUP
Base - BayesB
Procedure

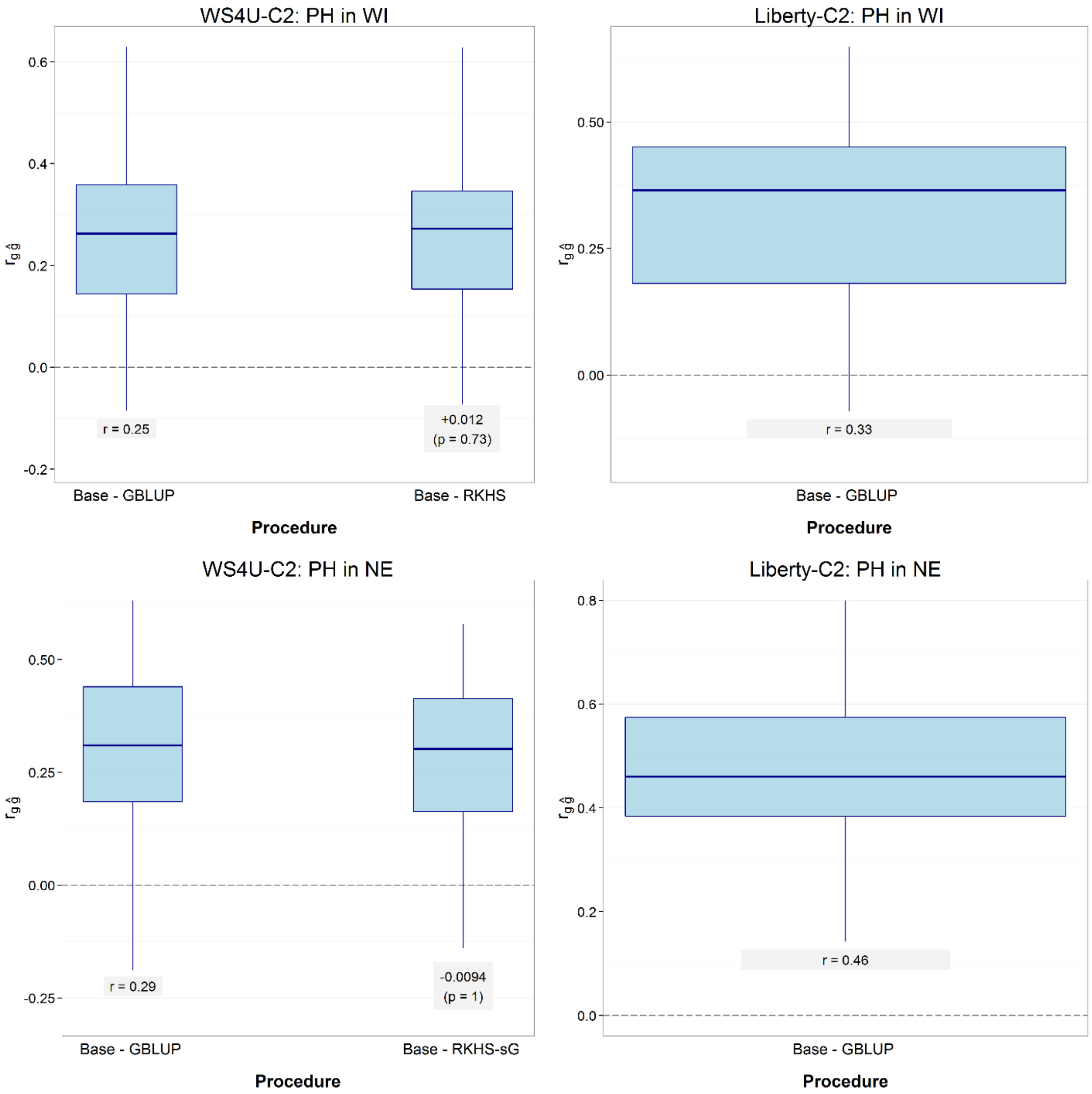
WS4U-C2: PH in WI
r = 0.25
+0.012
(p = 0.73)
Base - GBLUP
Base - RKHS
Procedure
Liberty-C2: PH in WI
r = 0.33
Base - GBLUP
Procedure
WS4U-C2: PH in NE
r = 0.29
-0.0094
(p = 1)
Base - GBLUP
Base - RKHS-sG
Procedure
Liberty-C2: PH in NE
r = 0.46
Base - GBLUP
Procedure

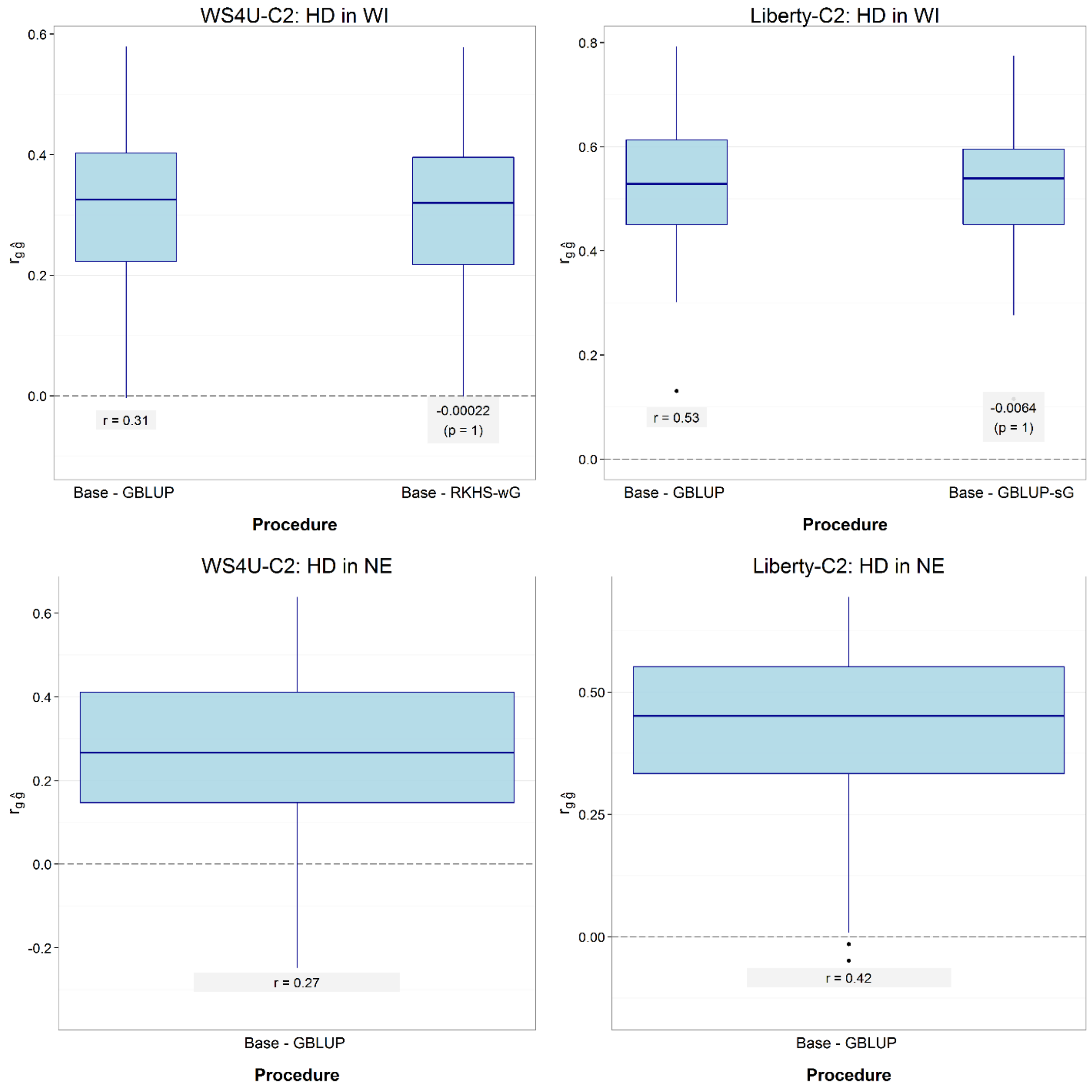

Figure S6. Validation of selected prediction procedures for all outcomes, ignoring all alternate marker-data transformations (i.e., other than Base). Prediction accuracies ($r_{g\hat{g}}$) were estimated with a within-population/within-environment learning scheme in five-fold cross-validation, replicated ten times. In each boxplot, the selected procedure (best prediction procedure according to non-replicated five-fold cross-validation; Tables 3 and S3) is compared to the standard procedure (Base – GBLUP). The significance of differences in prediction accuracies was assessed by two-sided paired Dunnett tests, which accounted for multiple testing of data transformations of prediction models. The t-statistics in Dunnett tests were adjusted to account for correlation among training sets in cross-validation, as described in Bouckaert and Frank (2004).
