# Supplementary material for: Accuracy of Genomic Prediction in Switchgrass (Panicum virgatum L.) Improved by Accounting for Linkage Disequilibrium
Source: G3 (Bethesda). 2016 Feb 10;6(4):1049–62. doi: 10.1534/g3.115.024950 (PMC4825640; doi:10.1534/g3.115.024950)
Supplement: Supplemental Material [file supp_g3.115.024950_TableS1.pdf]

Table S1. Results of association mapping for DMY in WI across both populations.

Gene	Feature	Chromosome	Position (bp)	MAF	R^2_{LR}
**Pavir.Ba03876**	intronic	Chr02a	77,527,751	0.097	0.42
**Pavir.Ga00641**	exonic	Chr07a	8,145,980	0.085	
**Pavir.Ha00300**	intronic	Chr08a	6,097,945	0.091	
**Pavir.Ia03558**	intronic	Chr09a	69,972,787	0.081	
**Pavir.Bb00675**	exonic	Chr02b	11,682,163	0.119	
**Pavir.Bb01690**	intronic	Chr02b	41,239,077	0.139	
**Pavir.Hb00247**	exonic	Chr08b	4,667,943	0.053	

Gene names and marker localizations are based on the reference genome sequencing and annotation of Panicum virgatum v1.1 (DOE-JGI, http://phytozome.jgi.doe.gov/). R^2_{LR}: likelihood-ratio-based R^2 statistic (Sun et al. 2010). Association mapping was performed using a linear mixed model with the outcome being DMY, PH or HD in WI or NE, with one fixed additive effect for a given marker assayed and a random background effect accounted for through a realized genetic relationship matrix derived from marker data as $\mathbf{K} \propto \mathbf{X}_{\mathrm{Base}}\mathbf{X}_{\mathrm{Base}}{}^{\mathrm{T}}$. The threshold used to declare significance of associations was a false discovery rate (as from Storey and Tibshirani 2003) lower than 0.05. The significant markers reported above were selected altogether in one linear mixed model, with a random background effect and fixed effects for markers, by forward stepwise selection based on the Bayesian information criterion. Significant associations were obtained only with DMY in WI (not with the eleven other outcomes).
