# Supplementary material for: Accuracy of Genomic Prediction in Switchgrass (Panicum virgatum L.) Improved by Accounting for Linkage Disequilibrium
Source: G3 (Bethesda). 2016 Feb 10;6(4):1049–62. doi: 10.1534/g3.115.024950 (PMC4825640; doi:10.1534/g3.115.024950)
Supplement: Supplemental Material [file supp_g3.115.024950_TableS2.pdf]

**Table S2.** Mean prediction accuracy across population and environment learning schemes for PH and HD in WI and NE.

| | | **PH in WI** | | **PH in NE** | |
|---|---|---|---|---|---|
| | | Within env. | Across env. | Within env. | Across env. |
| **WS4U-C2** | Within pop. | 0.254 | 0.253 | 0.292 | 0.297 |
| | Across pop. | 0.235 | 0.247 | 0.289 | 0.297 |
| **Liberty-C2** | Within pop. | 0.329 | 0.364 | 0.460 | 0.458 |
| | Across pop. | 0.334 | 0.367 | 0.461 | 0.454 |
| | | **HD in WI** | | **HD in NE** | |
| | | Within env. | Across env. | Within env. | Across env. |
| **WS4U-C2** | Within pop. | 0.312 | 0.314 | 0.268 | 0.285 |
| | Across pop. | 0.291 | 0.300 | 0.272 | 0.285 |
| **Liberty-C2** | Within pop. | 0.530 | 0.519 | 0.421 | 0.448 |
| | Across pop. | 0.506 | 0.478 | 0.410 | 0.431 |

Prediction accuracies were estimated with Base – GBLUP in five-fold cross-validation replicated ten times. The significance of differences in prediction accuracy was assessed by two-sided paired Dunnett tests, which accounted for multiple comparisons of learning schemes to a single reference (the within-population/within-environment scheme). The t-statistics in Dunnett tests were adjusted to account for correlation among training sets in cross-validation, as described in Bouckaert and Frank (2004). For a given population and trait-location combination, differences in prediction accuracy compared to the within- population/within-environment scheme were never deemed significant ($p > 0.10$ in paired Dunnett tests).
