# Supplementary material for: Accuracy of Genomic Prediction in Switchgrass (Panicum virgatum L.) Improved by Accounting for Linkage Disequilibrium
Source: G3 (Bethesda). 2016 Feb 10;6(4):1049–62. doi: 10.1534/g3.115.024950 (PMC4825640; doi:10.1534/g3.115.024950)
Supplement: Supplemental Material [file supp_g3.115.024950_TableS3.pdf]

**Table S3 -** Mean prediction accuracy across marker-data transformations and statistical models for PH and HD in WI and NE.

**PH in WI**

| | | GBLUP | GBLUP-wG | GBLUP-sG | RKHS | RKHS-wG | RKHS-sG | BayesA | BayesB | RF | (Mean) |
|---|---|---|---|---|---|---|---|---|---|---|---|
| **WS4U-C2** | **Base** | 0.298 | 0.291 | 0.238 | 0.303 | 0.291 | 0.213 | 0.300 | 0.302 | 0.086 | 0.258 |
| | **PCA** | 0.298 | 0.082 | 0.040 | 0.303 | 0.106 | 0.078 | 0.196 | 0.170 | 0.194 | 0.163 |
| | **Cor** | 0.289 | 0.259 | 0.153 | 0.279 | 0.263 | 0.205 | 0.292 | 0.306 | **0.313** | 0.262 |
| | **LD** | 0.288 | 0.302 | 0.196 | 0.293 | 0.291 | 0.212 | 0.286 | 0.294 | 0.064 | 0.247 |
| | **(Mean)** | 0.293 | 0.234 | 0.157 | 0.2945 | 0.238 | 0.177 | 0.269 | 0.268 | 0.164 | 0.233 |
| **Liberty-C2** | **Base** | 0.344 | 0.315 | 0.236 | 0.319 | 0.299 | 0.136 | 0.322 | 0.315 | 0.132 | 0.269 |
| | **PCA** | 0.344 | 0.195 | 0.085 | 0.319 | 0.24 | 0.141 | 0.328 | 0.344 | -0.046 | 0.217 |
| | **Cor** | 0.299 | 0.249 | 0.225 | 0.282 | 0.253 | 0.203 | 0.277 | 0.267 | 0.270 | 0.258 |
| | **LD** | **0.371** | 0.320 | 0.196 | 0.348 | 0.315 | 0.187 | 0.361 | 0.37 | 0.173 | 0.293 |
| | **(Mean)** | 0.340 | 0.270 | 0.186 | 0.317 | 0.277 | 0.167 | 0.322 | 0.324 | 0.132 | 0.259 |

**PH in NE**

| | | GBLUP | GBLUP-wG | GBLUP-sG | RKHS | RKHS-wG | RKHS-sG | BayesA | BayesB | RF | (Mean) |
|---|---|---|---|---|---|---|---|---|---|---|---|
| **WS4U-C2** | **Base** | 0.326 | 0.312 | 0.321 | 0.326 | 0.320 | 0.328 | 0.320 | 0.326 | 0.127 | 0.301 |
| | **PCA** | 0.326 | 0.130 | 0.130 | 0.326 | 0.159 | 0.159 | 0.198 | 0.188 | 0.187 | 0.200 |
| | **Cor** | 0.372 | 0.356 | 0.360 | 0.374 | 0.365 | 0.385 | 0.386 | **0.396** | 0.288 | 0.365 |
| | **LD** | 0.290 | 0.280 | 0.227 | 0.309 | 0.295 | 0.269 | 0.291 | 0.291 | 0.003 | 0.251 |
| | **(Mean)** | 0.329 | 0.270 | 0.260 | 0.334 | 0.285 | 0.285 | 0.299 | 0.300 | 0.151 | 0.279 |
| **Liberty-C2** | **Base** | **0.500** | 0.482 | 0.418 | 0.469 | 0.466 | 0.295 | 0.500 | 0.481 | 0.381 | 0.444 |
| | **PCA** | 0.500 | 0.377 | 0.377 | 0.469 | 0.398 | 0.398 | 0.496 | 0.499 | 0.237 | 0.417 |
| | **Cor** | 0.474 | 0.456 | 0.410 | 0.453 | 0.449 | 0.417 | 0.473 | 0.470 | 0.379 | 0.442 |
| | **LD** | 0.477 | 0.464 | 0.426 | 0.453 | 0.452 | 0.317 | 0.479 | 0.472 | 0.380 | 0.436 |
| | **(Mean)** | 0.488 | 0.445 | 0.408 | 0.461 | 0.441 | 0.357 | 0.487 | 0.481 | 0.344 | 0.435 |

| | | GBLUP | GBLUP-wG | GBLUP-sG | RKHS | RKHS-wG | RKHS-sG | BayesA | BayesB | RF | (Mean) |
|---|---|---|---|---|---|---|---|---|---|---|---|
| **HD in WI** | | | | | | | | | | | |
| **WS4U-C2** | **Base** | 0.254 | 0.262 | 0.207 | 0.271 | 0.271 | 0.224 | 0.271 | 0.217 | 0.129 | <u>0.234</u> |
| | **PCA** | 0.254 | 0.070 | 0.041 | 0.271 | 0.114 | 0.091 | 0.204 | 0.179 | 0.008 | 0.137 |
| | **Cor** | 0.224 | 0.200 | 0.101 | 0.232 | 0.209 | 0.094 | 0.212 | 0.224 | 0.157 | 0.184 |
| | **LD** | 0.234 | 0.247 | 0.234 | 0.249 | 0.257 | 0.238 | **<u>0.280</u>** | 0.192 | 0.035 | 0.218 |
| | **(Mean)** | 0.242 | 0.195 | 0.146 | 0.256 | 0.213 | 0.162 | 0.242 | 0.203 | 0.082 | 0.193 |
| **Liberty-C2** | **Base** | 0.577 | 0.598 | 0.599 | 0.581 | 0.595 | 0.587 | 0.565 | 0.555 | 0.543 | <u>0.578</u> |
| | **PCA** | 0.577 | 0.446 | 0.352 | 0.581 | 0.509 | 0.428 | 0.570 | 0.569 | 0.373 | 0.489 |
| | **Cor** | 0.528 | 0.550 | **<u>0.604</u>** | 0.527 | 0.547 | 0.599 | 0.528 | 0.517 | 0.504 | 0.545 |
| | **LD** | 0.563 | 0.564 | 0.583 | 0.571 | 0.567 | 0.579 | 0.565 | 0.560 | 0.557 | 0.568 |
| | **(Mean)** | 0.561 | 0.540 | 0.535 | 0.565 | 0.555 | 0.548 | 0.557 | 0.550 | 0.494 | 0.545 |
| **HD in NE** | | | | | | | | | | | |
| | | **GBLUP** | **GBLUP-wG** | **GBLUP-sG** | **RKHS** | **RKHS-wG** | **RKHS-sG** | **BayesA** | **BayesB** | **RF** | **(Mean)** |
| **WS4U-C2** | **Base** | 0.246 | 0.233 | 0.230 | 0.239 | 0.232 | 0.240 | 0.193 | 0.226 | 0.051 | 0.210 |
| | **PCA** | 0.246 | 0.151 | 0.114 | 0.239 | 0.202 | 0.187 | 0.230 | 0.229 | 0.139 | 0.193 |
| | **Cor** | 0.212 | 0.179 | 0.161 | 0.230 | 0.197 | 0.161 | 0.214 | **<u>0.271</u>** | 0.269 | <u>0.210</u> |
| | **LD** | 0.213 | 0.158 | 0.162 | 0.208 | 0.166 | 0.195 | 0.213 | 0.216 | 0.084 | 0.179 |
| | **(Mean)** | 0.229 | 0.180 | 0.167 | 0.229 | 0.199 | 0.196 | 0.213 | 0.236 | 0.136 | 0.198 |
| **Liberty-C2** | **Base** | **<u>0.420</u>** | 0.413 | 0.351 | 0.396 | 0.398 | 0.295 | 0.406 | 0.399 | 0.369 | <u>0.383</u> |
| | **PCA** | 0.420 | 0.298 | 0.073 | 0.396 | 0.330 | 0.169 | 0.387 | 0.366 | 0.077 | 0.280 |
| | **Cor** | 0.368 | 0.358 | 0.331 | 0.353 | 0.354 | 0.345 | 0.356 | 0.360 | 0.321 | 0.350 |
| | **LD** | 0.403 | 0.397 | 0.343 | 0.376 | 0.383 | 0.328 | 0.400 | 0.409 | 0.382 | 0.380 |
| | **(Mean)** | 0.403 | 0.367 | 0.275 | 0.380 | 0.366 | 0.284 | 0.387 | 0.384 | 0.287 | 0.348 |

*Prediction accuracies were estimated with a within-population/within-environment learning scheme in five-fold cross-validation, with no replication. For a given population and outcome (trait-location combination), the highest average value across marker-data transformations is underlined; the highest value across prediction procedures is underlined and bolded.*
